# Growth Hormone–Insulin-like Growth Factor Axis and GDF-15 in Critical Illness: Implications for Survival Stratification

**DOI:** 10.3390/life16081245

**Published:** 2026-07-27

**Authors:** Ioannis Ilias, Chrysi Keskinidou, Georgios Poupouzas, Vasileios Issaris, Nikolaos S. Lotsios, Efthymia Botoula, Marinella Tzanela, Dimitra A. Vassiliadi, Stelios Kokkoris, Charikleia S. Vrettou, Alice G. Vassiliou, Ioanna Dimopoulou

**Affiliations:** 1 Department of Endocrinology, Hippokration Hospital, 11527 Athens, Greece; 2 1st Department of Critical Care Medicine, Evangelismos Hospital, School of Medicine, National and Kapodistrian University of Athens, 10676 Athens, Greece; chrysakes29@gmail.com (C.K.); poupouzas.gewr.kw@gmail.com (G.P.); vasilisiss@gmail.com (V.I.); nlotsios@med.uoa.gr (N.S.L.); stkokkoris@med.uoa.gr (S.K.); vrettou@hotmail.com (C.S.V.); alvass75@gmail.com (A.G.V.); idimo@otenet.gr (I.D.); 3 Department of Endocrinology, Diabetes and Metabolism, National Expertise Center for Rare Endocrine Diseases, Evangelismos Hospital, 10676 Athens, Greece; bclf2008@yahoo.gr (E.B.); mtzanel@med.uoa.gr (M.T.); dvassiliadi@evaggelismos-hosp.gr (D.A.V.)

**Keywords:** critical illness, GH–IGF axis, GDF-15, IGFBP-2, biomarker intercorrelation, partial correlation, hierarchical clustering, ICU mortality, pilot study, STROBE

## Abstract

**Background**: The growth hormone (GH)–insulin-like growth factor (IGF) axis is profoundly dysregulated in critical illness. GDF-15 and individual IGF-binding proteins (IGFBPs) have separately been proposed as prognostic biomarkers, but to our knowledge, no prior study has simultaneously characterized all major GH–IGF axis components and GDF-15 in the same critically ill cohort, precluding assessment of their joint intercorrelation structure. **Aim**: To provide the first simultaneous characterization of the intercorrelation structure among ten GH–IGF axis components and GDF-15 in a single ICU cohort, testing whether this structure is robust to adjustment for illness severity; and, secondarily, to describe admission discriminatory performance relative to APACHE II and SOFA. **Methods**: This was a prospective observational pilot study of 43 critically ill adults with admission (T01) measurement of ten GH–IGF axis biomarkers and longitudinal follow-up to day 15. Spearman correlations and hierarchical clustering characterized the admission intercorrelation structure; partial correlations adjusting for APACHE II and SOFA, and bootstrap confidence intervals, assessed robustness. Secondary analyses included the examination of admission discrimination (ROC/AUC), a leave-one-out cross-validated combined model, and longitudinal trajectories. All analyses were exploratory, hypothesis-generating, and unadjusted for multiple comparisons unless stated. **Results**: Hierarchical clustering identified a coherent cluster comprising GDF-15, IGFBP-1, IGFBP-2, and growth hormone-binding protein (GHBP), distinct from classical GH-resistance markers (GHR vs. healthy controls, GHR vs. admission) and from GH, IGF-1, acid-labile subunit (ALS), and IGFBP-3. Within this cluster, GDF-15 correlated with IGFBP-1 (ρ = 0.65, 95% bootstrap CI 0.44–0.78), IGFBP-2 (ρ = 0.50, CI 0.19–0.71), and GHBP (ρ = 0.47, CI 0.20–0.68); GDF-15 showed no correlation with classical GH-resistance markers. These correlations were essentially unchanged after adjusting for APACHE II or SOFA (partial ρ within 0.03–0.16 of unadjusted values), indicating the structure is not attributable to shared confounding by illness severity. This robustness extended to further adjustment for IL-6, age, BMI, and mechanical-ventilation duration, and results from all 45 pairwise T01 correlations were re-examined with Benjamini–Hochberg false-discovery-rate correction (9 of 11 nominally significant pairs retained q < 0.05). However, the GDF-15–GHBP correlation, unlike the GDF-15–IGFBP-1/IGFBP-2 correlations, attenuated substantially after adjustment for IL-6 and was not consistent across a brain-injury/non-brain-injury subgroup sensitivity analysis, indicating this specific link is less specific than the others. In secondary exploratory analyses, GDF-15 had the highest individual admission AUC (0.74) among biomarkers but was substantially outperformed by APACHE II (AUC 0.90) and SOFA (AUC 0.81); a combined GDF-15 + IGFBP-2 model did not improve on GDF-15 alone. **Conclusions**: This study identifies a severity-independent intercorrelation structure linking GDF-15 to inhibitory IGFBPs, distinct from classical GH-resistance signaling, in critically ill patients. Although the findings do not support any clinical application at this stage, further study in adequately powered, multicenter cohorts can be contemplated.

## 1. Introduction

Critical illness triggers profound neuroendocrine and metabolic responses, including catabolism, substrate redistribution, and endocrine dysregulation [1,2]. A central component is the onset of GH resistance—characterized by an increase in GH secretion with a concomitant reduction in circulating IGF-1 [3]. Growth hormone (GH) resistance during inflammation is primarily mediated by the pro-inflammatory cytokines IL-1, TNF, and IL-6, which suppress the GH–IGF-1 axis through distinct but complementary mechanisms [4]. TNF and IL-1 directly downregulate hepatic growth hormone receptor (GHR) expression, reducing cellular responsiveness to circulating GH, whereas IL-6 predominantly impairs post-receptor signaling [5]. These mechanisms lead to reduced IGF-1 synthesis, contributing to growth failure, muscle wasting, and metabolic dysfunction commonly observed in chronic inflammatory diseases. This acquired GH resistance has been associated with prolonged catabolism, impaired muscle anabolism, delayed weaning from mechanical ventilation, and a poor prognosis when sustained beyond the acute phase [3,6]. The temporal pattern of these changes is biphasic: an acute neuroendocrine stress response in the first days is followed by a more chronic insufficiency pattern, providing the physiological rationale for serial biomarker measurements across the ICU course [2]. It is important to note that this cytokine-driven route is not the only path to elevated stress biomarkers: acute brain injury elevates GDF-15 and related stress signals principally through a massive central catecholamine surge and direct tissue destruction rather than through the systemic cytokine cascade described above, a biologically distinct mechanism relevant to the present general ICU cohort (Section 2.1).

Circulating IGF-1 is predominantly carried in a 150-kDa ternary complex with IGFBP-3 and the acid-labile subunit (ALS), extending its half-life from minutes to approximately 20 h [7,8]. During systemic stress, proteolytic disruption of this complex, reduced hepatic IGF-1 production, and pro-inflammatory signaling decrease IGF-1 bioavailability while increasing the inhibitory binding proteins IGFBP-1 and IGFBP-2 [7,9]. Counterintuitively, GH treatment in critically ill patients unexpectedly increased mortality [10].

Growth Differentiation Factor 15 (GDF-15), a divergent TGF-β superfamily member, is a pan-stress biomarker of mitochondrial dysfunction, hypoxic injury, and inflammation [11]. As a stress-induced mitokine, it suppresses the GH–IGF-1 axis by inhibiting hepatic JAK2/STAT5 signaling [12,13]. Normally, GH activates the GHR-JAK2-STAT5 pathway to promote hepatic IGF-1 transcription; elevated GDF-15, as observed in chronic diseases including heart failure and cancer, impairs JAK2/STAT5 phosphorylation, reducing IGF-1 production and inducing GH resistance. This may contribute to metabolic dysfunction and growth impairment, particularly in pediatric heart disease, where cardiac-derived GDF-15 suppresses the heart–liver endocrine axis and promotes failure to thrive. Circulating GDF-15 and IGF-1 are consistently inversely associated, linking mitochondrial stress and catabolic signaling to disruption of the GH/JAK2/STAT5/IGF-1 pathway. In heterogeneous ICU populations, including sepsis, GDF-15 concentrations at ICU admission correlate with organ dysfunction and independently predict short- and long-term mortality [14,15]. Serial measurements identify distinct trajectories, with persistently high GDF-15 associated with the poorest outcomes, while admission levels reflect cumulative disease burden [16]. GDF-15 also signals nutritional and metabolic stress [17] and is associated with muscle weakness in ICU survivors [18].

Although GDF-15 [14,15] and IGFBP-2 [19,20] have each been proposed as ICU prognostic biomarkers, no study, to our knowledge, has simultaneously assessed all major GH–IGF axis components, IGFBPs, and GDF-15 in the same cohort. This approach enables direct characterization of the complete axis and determination of whether GDF-15 forms part of the classical GH-resistance signature or represents a distinct stress signal that co-varies with inhibitory IGFBPs—questions that cannot be resolved by single-marker studies. Previously published analyses in this cohort examined longitudinal glucocorticoid receptor (GR) isoform expression, providing additional endocrine context [21].

The present study targets the GH–IGF axis/GDF-15 response to critical illness as a generic, etiology-independent metabolic-stress construct—not a sepsis-specific or trauma-specific signature—reflecting the case-mix of a general ICU. Diagnosis-specific effects are tested directly, rather than assumed to be absent, via a brain-injury-stratified sensitivity analysis reported in Section 3.7.

This prospective pilot study aimed to: (1) characterize the intercorrelation structure among all major GH–IGF axis components and GDF-15 at admission, and test whether this structure is robust to adjustment for illness severity (APACHE II, SOFA); (2) describe, as a secondary and explicitly exploratory analysis, individual biomarker discrimination for ICU mortality relative to APACHE II [22] and SOFA [23], and the performance of a combined GDF-15 + IGFBP-2 model under leave-one-out cross-validation; and (3) describe longitudinal trajectories of all biomarkers across four pre-specified timepoints. Given the small sample, all analyses are exploratory; none are intended, nor are they powered, to support clinical application.

## 2. Materials and Methods

### 2.1. Study Design, Setting, and Patients

This prospective observational pilot study was conducted in the 1st Department of Critical Care Medicine, Evangelismos Hospital, Athens, Greece—a mixed medical-surgical ICU that serves as a tertiary referral center for both general critical care. This dual function accounts for the high proportion of brain injury patients (51%), which reflects the unit’s referral pattern rather than deliberate enrichment and should be considered when assessing generalizability. Inclusion criteria were: consecutive adult patients (aged ≥18 years) admitted to the ICU. Exclusion criteria were: age under 18 years; an ICU stay shorter than 3 days (needed to ensure at least one follow-up biomarker sample beyond T01); brain death at admission; pregnancy; contagious disease (HIV, hepatitis); ICU readmission or transfer from another ICU; and prior or in-ICU corticosteroid administration before the first blood draw (chronic pre-admission therapy ≥ 1 mg/kg prednisone-equivalent for over a month, or any in-hospital corticosteroid administration prior to sampling). Furthermore, blood sampling was discontinued, and patients were subsequently withdrawn from the study, upon the administration of hydrocortisone for septic shock, ICU discharge, or death. Participant flow, enrolment, and sample availability at each timepoint are shown in Supplementary Figure S1. Patients with major burns are rarely admitted to our ICU. Individuals undergoing abdominal surgery were not included either because their ICU stay was very short (<3 days) or, in more severe cases such as peritonitis with septic shock, because corticosteroid therapy constituted an exclusion criterion. Cardiac surgery patients were likewise absent, as they are routinely managed in a dedicated cardiac ICU separate from our unit. Consequently, their non-representation in this cohort reflects the referral patterns and predefined exclusion criteria of the study rather than any deliberate omission.

Demographic data included age, sex, height, weight, and body mass index (BMI). Disease severity was quantified by APACHE II [22] and SOFA [23] at admission. The cohort comprised patients with brain injury (n = 22, 51%) and without (n = 21, 49%). Given only 9 non-survivors, the primary analyses pertain to the mixed cohort; nonetheless, Section 3.7 reports a diagnosis-stratified (brain-injury vs. non-brain-injury) sensitivity analysis and a non-survivor diagnosis-category breakdown as explicitly exploratory secondary observations, not as confirmatory subgroup findings. Primary outcome was ICU mortality. This endpoint was chosen a priori because the biological question under study (acute neuroendocrine/metabolic stress response) is most proximally linked to the acute critical-illness episode itself, whereas 28-day or hospital mortality also incorporate post-ICU events further removed from the T01–T04 biomarker measurement window; a 28-day mortality sensitivity analysis is reported in Section 3.7. The local ethics committee approved the study, and written informed consent was obtained from patients or their legal representatives (reg. no. 317/19 June 2024). The study was conducted and reported in accordance with STROBE guidelines for observational cohort studies [24].

### 2.2. Assays and Sample Collection

Venous blood samples were collected at four pre-specified timepoints aligned with the known biphasic temporal pattern of critical illness neuroendocrinology [2]: T01 (within 48 h of admission—acute stress phase), T02 (day 5 ± 1—early stabilization), T03 (day 10 ± 1—transition phase), and T04 (day 15 ± 1—late phase). Samples were centrifuged immediately after collection and serum stored at −80 °C until batch analysis at study end.

All biomarkers were measured using commercially available enzyme-linked assay (ELISA) kits validated for human serum. Specific assays: ALS (acid-labile subunit): Wuhan Fine Biotech Co., Ltd., Wuhan, China; detection limit 0.938 ng/mL; intra-assay CV 4.8%; inter-assay CV 5.6%. GHR (GH receptor serum protein concentration): Wuhan Fine Biotech Co. Ltd.; Wuhan, China, detection limit 0.469 ng/mL; intra-assay CV 4.7%; inter-assay CV 5.5%. IGFBP-1: Wuhan Elabscience Biotechnology Co., Ltd.; Wuhan, China, detection limit 0.1 ng/mL; intra-assay CV 5.5%; inter-assay CV 7.3%. IGFBP-2: Wuhan Elabscience Biotechnology Co., Ltd.; Wuhan, China, detection limit 0.08 ng/mL; intra-assay CV 4.7%; inter-assay CV 7.9%. IGFBP-3: Wuhan Elabscience Biotechnology Co., Ltd., Wuhan, China, detection limit 0.47 ng/mL; intra-assay CV 4.8%; inter-assay CV 8.2%. GDF-15: Wuhan Elabscience Biotechnology Co., Ltd.; Wuhan, China, detection limit 14.06 pg/mL; intra-assay CV 5.2%; inter-assay CV 6.9%. GH and IGF-1 were measured using validated immunoradiometric or immunofluorometric assay kits (Mediagnost GmbH, Reutlingen, Germany); GHBP by ELISA (R&D Systems, Minneapolis, MN, USA). All kits are validated for use with human serum. GH receptor (GHR) expression in peripheral blood mononuclear cells (PBMCs) was measured by quantitative RT-PCR. PBMCs were isolated by density-gradient centrifugation (Ficoll-Hypaque) immediately after blood collection. GHR expression is reported in two ways: relative to age- and sex-matched healthy controls (GHR vs. HC) and relative to each patient’s own T01 (admission) value (GHR vs. T01), capturing the intra-individual longitudinal change from admission onwards; by definition, GHR vs. T01 equals 1.0 for all patients at T01 and becomes informative at T02–T04 as intra-individual trajectories diverge. The healthy-control (HC) reference pool used for RT-PCR relative quantification of GHR expression (2^−ΔΔCt^ method, normalized to the HC baseline) comprises age- and sex-matched healthy blood donors, consistent with the reference population described for PBMC-based receptor-expression assays in this same patient cohort in our companion methodological report [21]. This HC comparator applies only to the GHR expression assay; absolute reference-range values for the other nine biomarkers in this manuscript were not part of the dataset available for the present analysis, which reports only the T01–T04 patient measurements for those markers. Regarding the potential confounding effect of blood transfusions on PBMC measurements: patients receiving transfusions within 48 h before any GHR blood draw were identified by medical record review. The proportion of transfused patients did not differ significantly between survivors and non-survivors.

### 2.3. Statistical Analysis

Analyses were performed in Python 3.12 (Python.org; accessed on 1 June 2026) and reported in accordance with STROBE guidelines [24]. All statistical tests are two-sided. No correction for multiple comparisons was applied; with ten biomarkers tested across multiple analytical approaches, all results must be treated as exploratory and hypothesis-generating. Continuous variables are reported as median (IQR); categorical variables as n (%). Admission biomarker comparisons used the two-sided Mann–Whitney U (MWU) test; categorical variables used Fisher’s exact test.

The primary analysis of this study was the characterization of the admission (T01) intercorrelation structure among all ten GH–IGF axis biomarkers. Spearman rank correlations were computed for all pairwise combinations, and hierarchical clustering (average linkage, distance = 1 − ρ) was applied to the resulting correlation matrix to identify coherent biomarker groupings without reliance on arbitrary significance thresholds. To assess whether the observed intercorrelation structure could be explained by shared confounding from illness severity, partial Spearman correlations adjusting for APACHE II and, separately, for SOFA were computed for the key biomarker pairs identified by clustering, using a rank-residual approach. Precision of key correlation estimates was further characterized using percentile bootstrap 95% confidence intervals (5000 resamples). As a further check on the 45 pairwise T01 correlations, Benjamini–Hochberg false-discovery-rate (FDR) q-values were computed across all pairs (Supplementary Table S3); q < 0.05 was considered FDR-significant.

Spearman correlations between individual biomarkers and APACHE II/SOFA scores were also computed as a secondary descriptive analysis, while individual biomarker discrimination for ICU mortality was assessed by ROC/AUC at T01 [25], alongside APACHE II [22] and SOFA [23]. A combined GDF-15 + IGFBP-2 logistic regression model (log-transformed, standardized inputs) was evaluated by leave-one-out cross-validation (LOO-CV) AUC [26]. Given that this cohort includes only 9 non-survivors, these analyses are reported as secondary observations only and are not intended to support any claim of clinical utility.

We restricted the primary analysis to correlation and hierarchical clustering, rather than multivariable outcome prediction, because only 9 events were observed. A model adjusting for even 3–4 covariates would provide fewer events per parameter than the ≥10 events-per-variable threshold generally considered necessary for stable logistic-regression estimates. In contrast, correlation and clustering use the full continuous biomarker distributions across all 43 patients without conditioning on the rare outcome and require no comparable events-per-parameter budget. Accordingly, these analyses were designated primary, while outcome-conditioned methods (ROC/AUC, LOO-CV) were restricted to an explicitly secondary, exploratory role. Longitudinal trajectories were additionally modelled using a GEE-approximation with cluster-robust sandwich variance estimator (clustered by patient), applied to log-transformed biomarker values, and are reported in full in Supplementary Table S2 and Supplementary Figure S1. This analysis is exploratory, because reliable cluster-robust variance estimation requires far more clusters than the declining non-survivor group provides (9 at T01 to 3 at T04) and because informative missingness from early death/discharge biases later-timepoint estimates. Full methodological detail (variance-estimator specification, cluster-count rationale, and the ≥2-timepoint sensitivity restriction) is given in Supplementary Methods, with results in Supplementary Table S2.

## 3. Results

**Figure 1:**
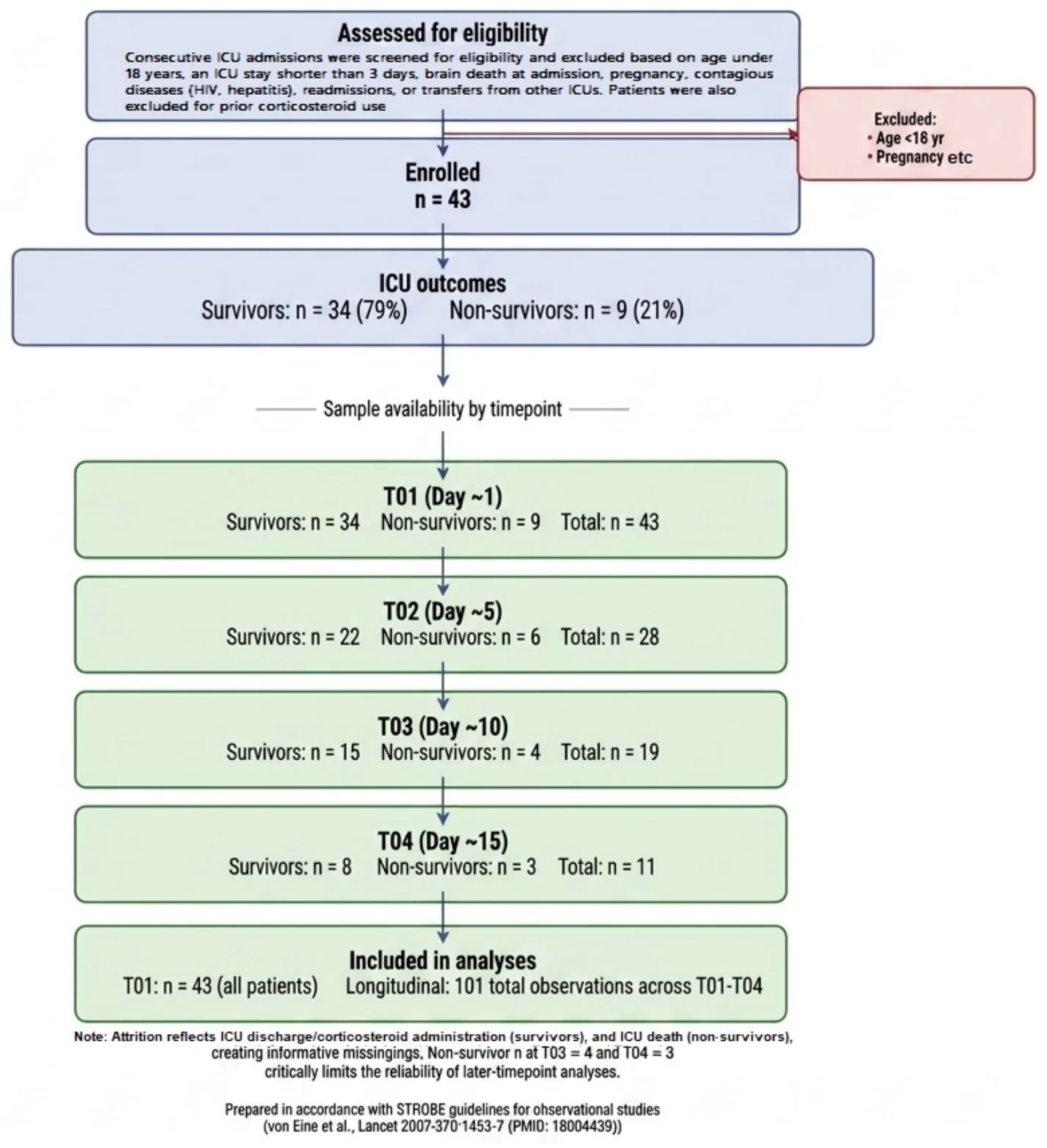
STROBE-compliant [24] participant flow diagram showing enrolment, ICU outcomes, and sample availability at each timepoint (T01–T04). Attrition reflects ICU discharge (survivors) and death (non-survivors), creating informative missingness at later timepoints; non-survivor n at T03 and T04 (4 and 3, respectively) limits the reliability of later-timepoint analyses (see Section 2.3, Supplementary Methods).

Forty-three critically ill patients were enrolled: 34 survivors and 9 non-survivors. Participant flow, enrolment, and sample availability at each timepoint are shown in Figure 1. Baseline characteristics are shown in Table 1. Median age was 58 years (IQR 51–68); 42% were female. Brain injury was the leading diagnosis (51%), reflecting the general ICU care function of the unit; sepsis accounted for 23%. Mechanical ventilation was required in 70% at T01.

Non-survivors had markedly greater illness severity: median APACHE II 18 (IQR 17–25) vs. 10 (IQR 7–16; *p* < 0.001), median SOFA 9 (IQR 8–11) vs. 6 (IQR 2–9; *p* = 0.005), and longer MV duration (median 19 vs. 6 days; *p* = 0.007). Age, sex, BMI, diagnosis, and MV status at T01 did not differ nominally between groups. All *p*-values are exploratory and unadjusted.

### 3.1. Admission Biomarkers and ICU Survival

Admission biomarker concentrations, Hodges–Lehmann effect sizes, and significance testing are presented in Table 2. To avoid relying on a single asymptotic test at this sample size, two-sided *p*-values were computed using both the Mann–Whitney U test and a label-permutation test (10,000 resamples on log-transformed values), which does not rely on assumptions regarding the underlying sampling distribution. The two approaches agreed closely for every biomarker (Table 2), supporting the robustness of the nominal findings to choice of test. No GH–IGF axis component differed nominally significantly between groups at T01 (all *p* > 0.05). IGFBP-2 showed a borderline trend (*p* = 0.054, permutation *p* = 0.059). GDF-15 was the only biomarker to reach nominal statistical significance by both tests (MWU *p* = 0.030; permutation *p* = 0.027; not corrected for 10 simultaneous comparisons): non-survivors had higher median concentrations (12.96 ng/mL, IQR 5.34–31.16) than survivors (5.97 ng/mL, IQR 2.14–11.78), a Hodges–Lehmann shift of 8.37 ng/mL (95% bootstrap CI 0.63–65.84—wide, but excluding zero). Partially overlapping IQRs and the wide effect-size CI are consistent with AUC 0.74 rather than complete separation. GDF-15 had the highest individual biomarker AUC (0.74), followed by IGFBP-2 (0.71) and GHBP (0.64); the remaining biomarkers had AUCs 0.53–0.60.

### 3.2. Admission Concentrations of GDF-15 and IGFBP-2

**Figure 2:**
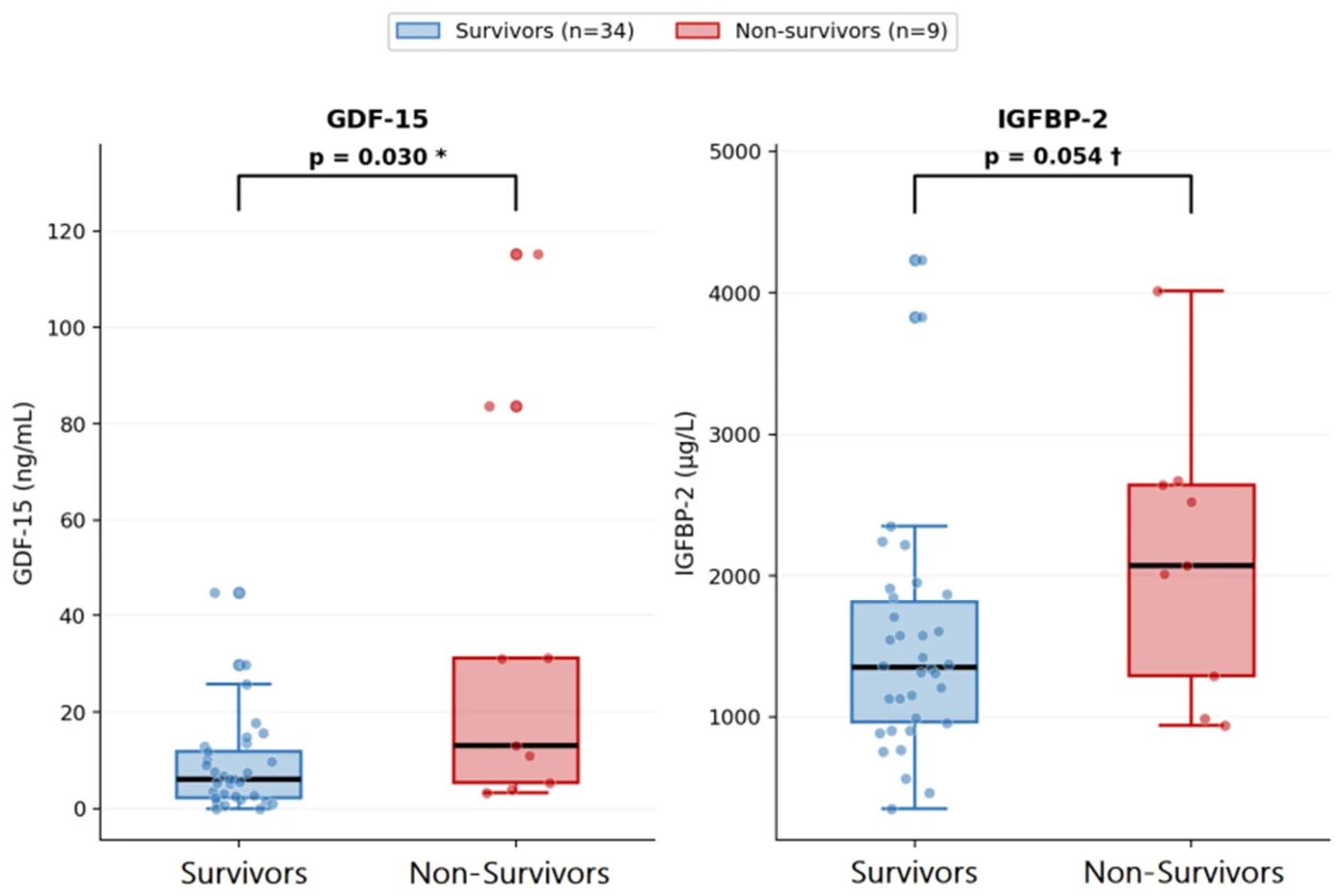
Admission (T01) concentrations of GDF-15 (ng/mL) and IGFBP-2 (µg/L) in survivors (blue, n = 34) and non-survivors (red, n = 9). Boxes: IQR; line: median; whiskers: 1.5 × IQR; dots: individual values (jittered). MWU *p*-values above brackets. * Nominally significant (unadjusted *p* < 0.05; not corrected for 10 simultaneous comparisons). † Borderline (*p* < 0.10). IQR overlap is consistent with AUC 0.74 rather than complete separation.

Figure 2 shows admission (T01) concentrations of GDF-15 and IGFBP-2 as box plots with individual data points. GDF-15 and IGFBP-2 concentrations by survival status correspond to the values already reported in Section 3.1 and Table 2; the overlapping IQRs visible here for both markers are consistent with their respective AUCs (0.74 and 0.71) rather than complete group separation.

### 3.3. Intercorrelation Structure Among T01 Biomarkers—Principal Analysis

**Figure 3:**
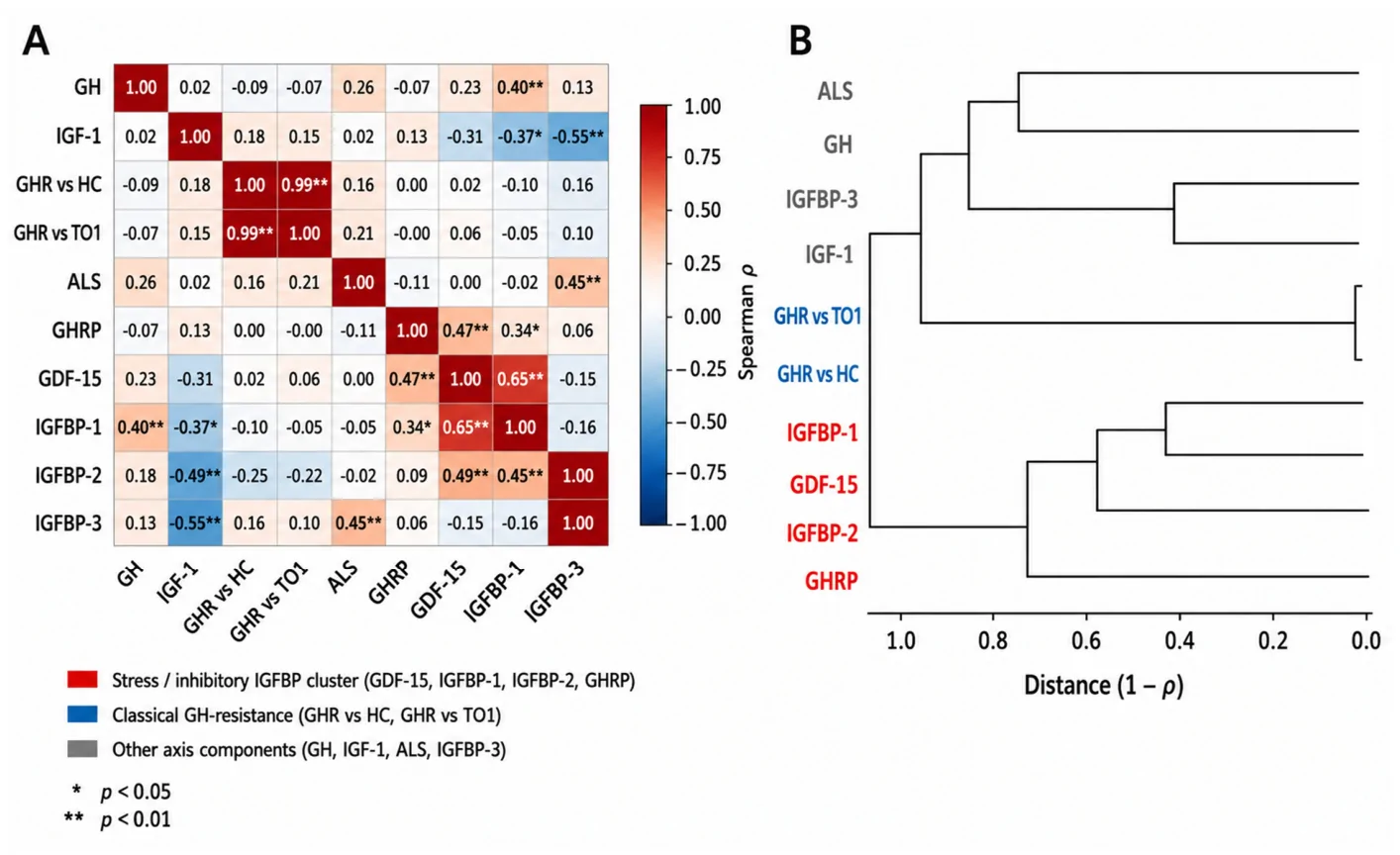
(**A**) Spearman rank correlation matrix for all ten T01 biomarkers (n = 40–43). Positive correlations in red, negative in blue. Cell values: Spearman ρ; * *p* < 0.05, ** *p* < 0.01. (**B**) Hierarchical clustering (average linkage) of the same correlation matrix using distance = 1 − ρ, shown as a dendrogram. Leaf labels are colored by cluster membership at k = 3: red = GDF-15/inhibitory IGFBP/GHBP cluster; blue = classical GH-resistance markers; grey = remaining axis components. All *p*-values are unadjusted and exploratory; the clustering itself does not depend on *p*-value thresholds.

Because this dataset measures all major GH–IGF axis components and GDF-15 simultaneously in the same critically ill cohort, it is well positioned to address a question that single-marker studies cannot: does GDF-15 behave as part of the classical GH-resistance signature, or as a separate stress signal that happens to co-vary with the inhibitory IGFBPs? Figure 3A shows the full Spearman correlation matrix for all ten T01 biomarkers (all *p*-values unadjusted and exploratory). GDF-15 correlated nominally significantly with IGFBP-1 (ρ = 0.65, *p* < 0.001), IGFBP-2 (ρ = 0.50, *p* = 0.001), and GHBP (ρ = 0.47, *p* = 0.002), and showed a non-significant trend toward inverse correlation with IGF-1 (ρ = −0.31, *p* = 0.053). GDF-15 did not correlate nominally with GH, ALS, GHR vs. HC, or GHR vs. T01—the classical GH-resistance markers. IGF-1 showed nominally significant inverse correlations with IGFBP-2 (ρ = −0.49) and IGFBP-1 (ρ = −0.37).

To characterize this structure without relying on an arbitrary significance threshold for each of the 45 pairwise comparisons, hierarchical clustering (average linkage, distance = 1 − ρ) was applied to the full correlation matrix (Figure 3B). This unsupervised approach, which uses no *p*-values and is not subject to multiple-comparison concerns in the same way, identified three coherent groupings: (1) a cluster comprising GDF-15, IGFBP-1, IGFBP-2, and GHBP; (2) the classical GH-resistance markers GHR vs. HC and GHR vs. T01, which are themselves nearly collinear (ρ = 0.99) by construction; and (3) a remaining group comprising GH, IGF-1, ALS, and IGFBP-3. GDF-15 clustered specifically with the inhibitory IGFBPs and GHBP—not with the classical GH-resistance markers—providing independent, threshold-free confirmation of the pattern seen in the correlation matrix.

### 3.4. Robustness of the Intercorrelation Structure to Illness Severity

**Figure 4:**
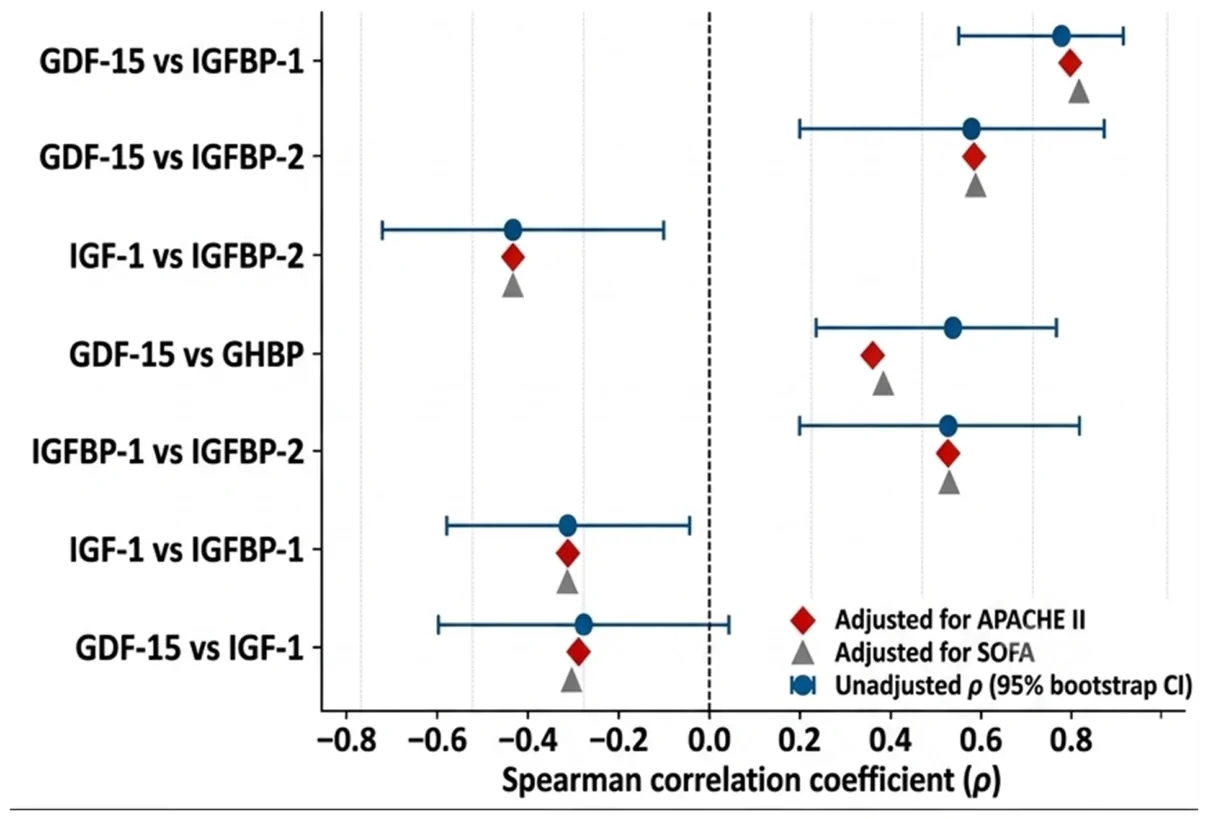
Key biomarker intercorrelations: unadjusted Spearman ρ with 95% bootstrap percentile confidence intervals (blue circles; 5000 resamples), compared with partial Spearman ρ adjusting for APACHE II (red diamonds) and for SOFA (grey triangles). Pairs are ordered by absolute unadjusted ρ. The close agreement between unadjusted and adjusted estimates indicates the intercorrelation structure is not attributable to shared confounding by illness severity.

A natural concern is whether the GDF-15–IGFBP intercorrelation simply reflects shared confounding by illness severity, since both GDF-15 and several IGFBPs are themselves associated with APACHE II and SOFA (Section 3.5). To address this directly, partial Spearman correlations adjusting for APACHE II, and separately for SOFA, were computed for the key pairs identified by clustering (Figure 4, Table 3). If the intercorrelation were driven mainly by shared severity confounding, adjustment should substantially attenuate the correlations toward zero. This was not observed. The GDF-15–IGFBP-1 correlation was ρ = 0.65 unadjusted and ρ = 0.67 (APACHE II-adjusted)/ρ = 0.69 (SOFA-adjusted); GDF-15–IGFBP-2 was ρ = 0.50 unadjusted vs. 0.50/0.50 adjusted; GDF-15–GHBP was ρ = 0.47 unadjusted vs. 0.31/0.34 adjusted (the largest attenuation observed, though the correlation remained nominally significant in both cases). Across all seven pairs tested, adjustment changed ρ by no more than 0.16 in either direction, and every pair that was nominally significant unadjusted remained nominally significant after adjustment for either score. This indicates that the GDF-15–inhibitory-IGFBP intercorrelation structure is not simply attributable to shared illness-severity confounding, and supports the hypothesis that these markers share upstream regulatory drivers beyond generic severity.

### 3.5. Correlations with Illness Severity

Spearman rank correlations between individual biomarkers and clinical severity scores are shown in Table 4 (all unadjusted). GH–IGF axis components showed no nominally significant correlations with APACHE II or SOFA. GHBP showed nominally significant positive correlations with both (APACHE II: ρ = 0.476, *p* = 0.001; SOFA: ρ = 0.452, *p* = 0.003). GDF-15 showed the strongest severity correlations (APACHE II: ρ = 0.501, *p* = 0.001; SOFA: ρ = 0.455, *p* = 0.002). IGFBP-2 did not correlate nominally with either score (APACHE II: ρ = 0.102; SOFA: ρ = 0.086), suggesting its prognostic information may be partially independent of measured severity—consistent with its retained correlation with GDF-15 after severity adjustment (Section 3.4).

### 3.6. Secondary, Exploratory Analyses: Discrimination and Longitudinal Trajectories

The analyses in this section are presented as secondary and exploratory; they are not intended to support any notion of predictive performance or clinical utility. They are included for completeness and to contextualize the correlation findings above against established severity scores.

#### 3.6.1. Admission Discrimination and Cross-Validated Combined Model

**Figure 5:**
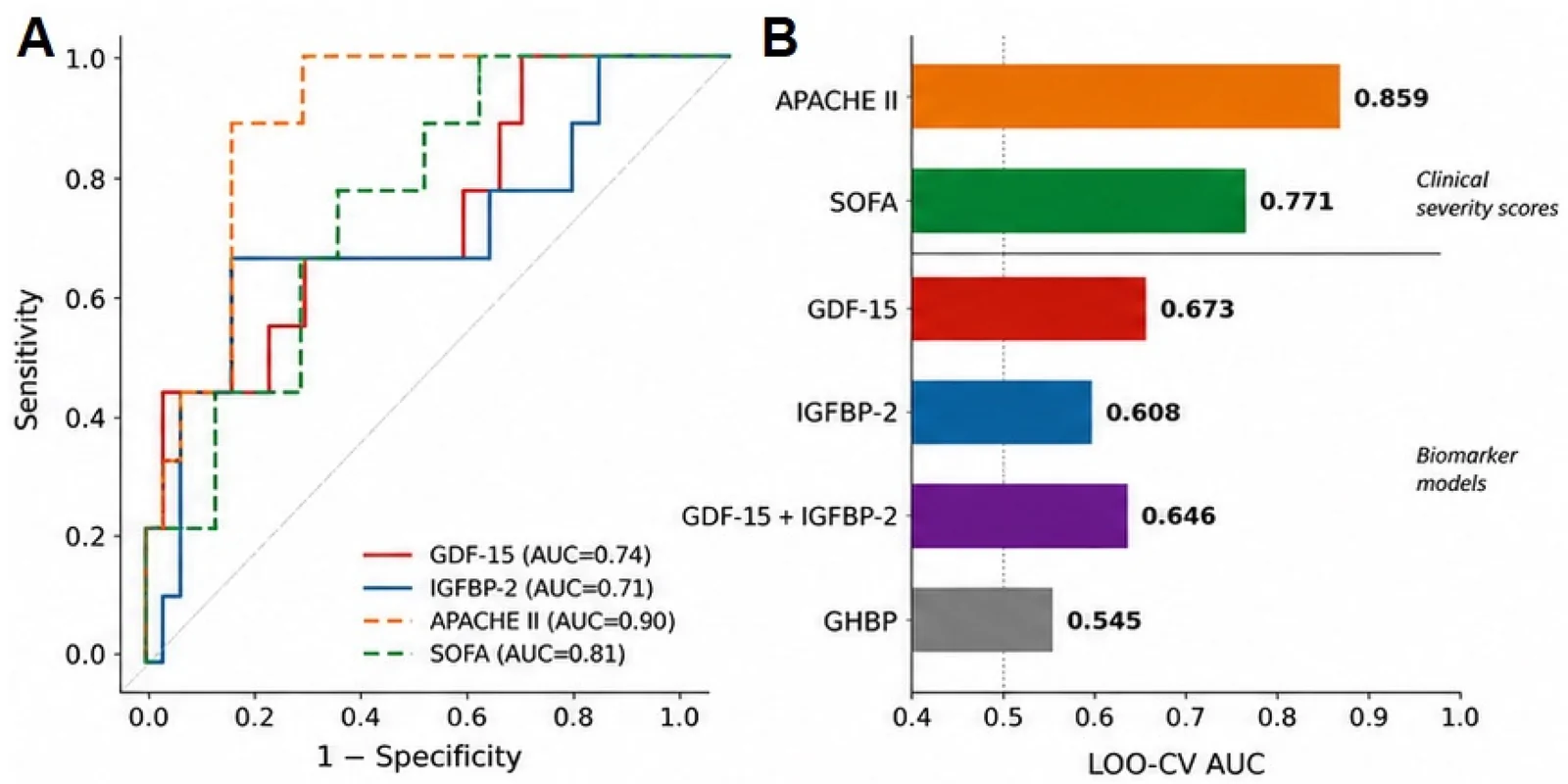
Secondary, exploratory discrimination analyses for ICU mortality (n = 43; 9 non-survivors). (**A**) ROC curves at T01 for the two top biomarkers (GDF-15, IGFBP-2) and clinical severity scores (APACHE II, SOFA); dotted diagonal = chance. (**B**) Leave-one-out cross-validated (LOO-CV) AUC for individual biomarkers, a combined GDF-15 + IGFBP-2 model, and clinical scores. APACHE II and SOFA substantially outperform all biomarker-based models. All estimates are exploratory, derived from the full analysis sample without external validation. With 9 events, AUC estimates carry wide confidence intervals not depicted here; inclusion is to contextualize biomarkers against severity scores, not to propose diagnostic/prognostic use.

Figure 5A presents ROC curves for the two top-performing biomarkers (GDF-15, IGFBP-2) alongside APACHE II and SOFA; curves for the remaining eight biomarkers (AUC 0.53–0.64) are omitted from the main figure for clarity and are reported in full in Table 2. APACHE II (AUC 0.90) and SOFA (AUC 0.81) substantially outperformed all biomarkers, as expected for validated multivariable severity scores [22,23]. With only 9 events, these AUC point estimates carry very wide confidence intervals; their purpose here is to contextualize the correlation findings against established severity scores, not to propose the biomarkers as diagnostic or prognostic tests. Figure 5B shows leave-one-out cross-validated (LOO-CV) AUCs: APACHE II (0.859) and SOFA (0.771) again substantially outperformed all biomarker-based models. Among biomarkers, GDF-15 alone had the highest LOO-CV AUC (0.67); a combined GDF-15 + IGFBP-2 logistic model achieved a LOO-CV AUC of 0.65—lower than GDF-15 alone. The 0.04 numerical difference between this combined model and IGFBP-2 alone (0.61) is within sampling variability at n = 9 events (with no meaningful additive predictive value) [26]. Indeed, given that GDF-15 and IGFBP-2 are themselves correlated (ρ = 0.50, Table 2), a combined model failing to outperform its stronger single component is close to a statistical tautology rather than a novel negative finding. These discrimination estimates are derived from the same 43-patient sample used for all analyses, are not externally validated, and should not be interpreted as evidence of clinical utility.

#### 3.6.2. Longitudinal Trajectories

Longitudinal trajectories of all ten biomarkers across T01–T04 are presented in Supplementary Figure S1 and Supplementary Table S2, rather than in the main text, given the cluster-count and informative-missingness limitations detailed in Section 2.3 and Supplementary Methods. As a transparency check, a label-permutation test comparing each patient’s early (T01–T02) versus late (T03–T04) mean log-biomarker value found that the GDF-15 divergence signal reversed direction in the restricted n = 18 subsample with both early and late measurements (permutation p = 0.17)—opposite to the robust admission-level finding (Section 3.1 and Section 3.2). This instability is informative: longitudinal or trajectory-based findings from this dataset should not be considered confirmatory. IGFBP-2, by contrast, showed a more consistent early-to-late divergence (permutation p = 0.003) and may be a more promising candidate for longitudinal follow-up in a larger, prospectively designed cohort.

### 3.7. Sensitivity to Additional Confounders, Diagnostic Subgroup, and Relationship to Systemic Inflammatory Markers

To address residual confounding, cohort heterogeneity, and the relationship of the principal biomarker cluster to standard inflammatory markers, we conducted a set of sensitivity analyses summarized here and detailed fully in Supplementary Table S3.

Extended confounder adjustment: In addition to the APACHE II- and SOFA-adjusted partial correlations reported in Section 3.4, we computed partial Spearman correlations for the four key pairs adjusting for IL-6, for IL-6 together with APACHE II, for age and BMI jointly, and for mechanical-ventilation duration (Table 5). The GDF-15–IGFBP-1 and GDF-15–IGFBP-2 correlations were essentially unchanged across every adjustment tested (partial ρ within 0.03–0.16 of the unadjusted value in all cases), as was the IGFBP-1–IGFBP-2 correlation. The GDF-15–GHBP correlation behaved differently: it remained robust to adjustment for age, BMI, and mechanical-ventilation duration, but attenuated substantially and lost nominal significance after adjustment for IL-6 (ρ 0.47 → 0.24, *p* = 0.13) or IL-6 plus APACHE II (ρ → 0.21, *p* = 0.20). We also re-examined all 45 pairwise T01 correlations with Benjamini–Hochberg FDR correction: 9 of 11 nominally significant pairs (*p* < 0.05) retained q < 0.05, including all three GDF-15–inhibitory-marker pairs and IGFBP-1–IGFBP-2; the two exceptions (IGF-1–IGFBP-1, GHBP–IGFBP-1) are flagged as not FDR-significant in Supplementary Table S3.

**Figure 6:**
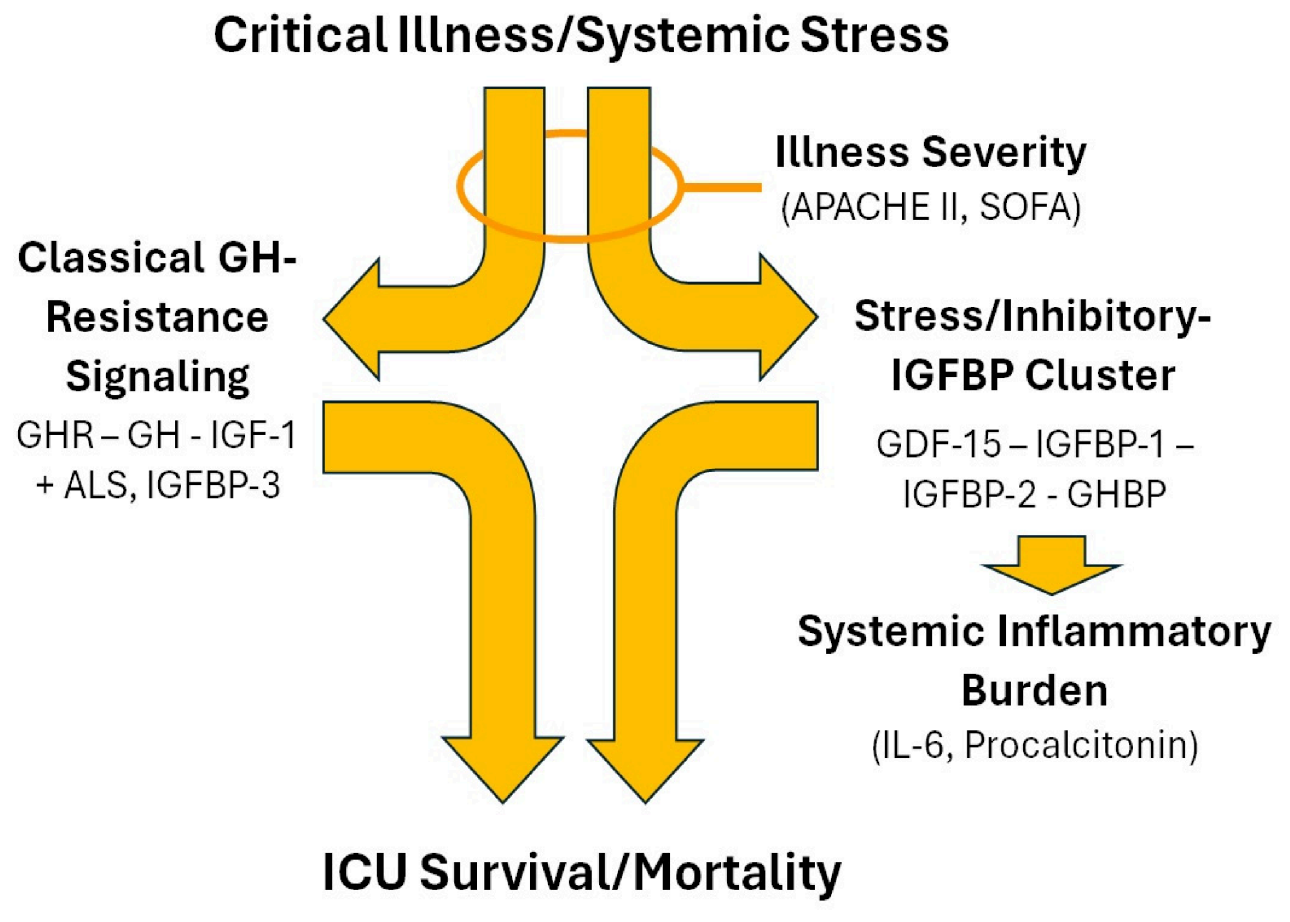
Schematic summary of the proposed relationships among critical illness, the classical GH-resistance axis, the stress/inhibitory-IGFBP cluster, systemic inflammation, and ICU mortality, integrating the sensitivity analyses in Section 3.7. Arrows denote statistical association observed in this dataset, not established causal direction; all relationships are exploratory, cross-sectional, and hypothesis-generating. GDF-15-IGFBP-1/GDF-15-IGFBP-2 correlations persist after adjustment for APACHE II, SOFA, age, BMI, mechanical-ventilation duration, and IL-6 (partial *p* within 0.03–0.11 of unadjusted values). GDF-15-GHBP correlation attenuates substantially after adjustment for IL-6 (*p* 0.47–0.24. *p* −0.13), suggesting this specific link is partly explained by generic inflammatory burden. The two clusters (classical GH-resistance vs. stress/inhibitory-IGFBP) show no nominally significant cross-cluster correlation, arguing against a single unified GH-resistance signal.

Relationship to IL-6, procalcitonin, and lactate: GDF-15 correlated strongly with IL-6 (ρ = 0.76, *p* < 0.001) and moderately with procalcitonin (ρ = 0.45, *p* = 0.01), but not significantly with maximum lactate (ρ = 0.24, *p* = 0.13). As detailed above, the GDF-15–IGFBP-1/IGFBP-2 links survive IL-6 adjustment essentially intact, arguing against these two links being a simple echo of inflammation; the GDF-15–GHBP link, by contrast, is substantially explained by shared IL-6-associated inflammatory burden. Thus, the inhibitory-IGFBP arm of the cluster (GDF-15–IGFBP-1, GDF-15–IGFBP-2, IGFBP-1–IGFBP-2) appears more IL-6-independent and specific than the GDF-15–GHBP link (Figure 6).

Diagnostic-subgroup sensitivity: When stratified by admission diagnosis into brain injury (n = 22) versus non-brain-injury (n = 21), the GDF-15–IGFBP-1 correlation was strong and significant in both strata (brain injury: ρ = 0.75, *p* < 0.001; non-brain-injury: ρ = 0.63, *p* = 0.002). The GDF-15–IGFBP-2 and IGFBP-1–IGFBP-2 correlations, however, were present only in the non-brain-injury stratum (ρ = 0.63, *p* = 0.002 and ρ = 0.48, *p* = 0.03, respectively) and were weak and non-significant among brain-injury patients (ρ = 0.29, *p* = 0.20 and ρ = 0.14, *p* = 0.54). This cluster is therefore not uniformly present across diagnostic subgroups at this sample size; the GDF-15–IGFBP-1 relationship appears to be the most diagnosis-independent component, while GDF-15–IGFBP-2 and IGFBP-1–IGFBP-2 should be regarded as provisional pending replication in a larger, diagnosis-balanced cohort.

Non-survivor diagnosis category and sepsis source: A formally adjudicated proximate cause of death was not part of this study’s data collection and was not reconstructed retrospectively. As a data-supported proxy, the primary admission diagnoses of the 9 non-survivors were as follows: 5/9 (56%) primarily neurological/traumatic (subarachnoid or intraventricular hemorrhage, or polytrauma with head injury) and 4/9 (44%) non-neurological, all four with documented sepsis during the ICU stay. Of the 10 sepsis patients overall, a documented infection source was available for 4/10 (respiratory, intra-abdominal, cholecystitis, and one confirmed Staphylococcus aureus bacteremia with a respiratory co-source); source was not systematically documented for the remaining 6/10, and infective endocarditis was not part of a systematic screening protocol for this biomarker study, so a cohort-wide rate cannot be reported.

28-day mortality sensitivity: Because ICU mortality (Section 2.1) is influenced by discharge practice, we repeated the GDF-15 discrimination analysis using 28-day mortality (5 events). GDF-15 AUC was 0.65 for 28-day mortality versus 0.74 for ICU mortality (Mann–Whitney *p* = 0.28 vs. 0.03)—directionally consistent but weaker and non-significant, as expected with fewer, more temporally distant events; this endpoint sensitivity should be considered alongside the primary ICU-mortality results.

Taken together, these analyses strengthen confidence in the GDF-15–IGFBP-1 relationship specifically, refine the GDF-15–GHBP relationship as substantially IL-6-mediated, and identify diagnosis-heterogeneity as a genuine, data-demonstrated limitation for the GDF-15–IGFBP-2 and IGFBP-1–IGFBP-2 links. A schematic summary integrating these relationships is provided in Figure 6 (Discussion).

## 4. Discussion

This prospective pilot study provides a simultaneous characterization of all major GH–IGF axis components together with GDF-15 in a single critically ill cohort. The principal finding is that GDF-15 forms a coherent, severity-independent intercorrelation cluster with the inhibitory binding proteins IGFBP-1 and IGFBP-2 and with GHBP, while remaining distinct from the classical GH-resistance markers GHR (vs. healthy controls and vs. admission), GH, ALS, and IGFBP-3. This structure was robust to hierarchical clustering, a method that does not rely on arbitrary significance thresholds, and was essentially unchanged after adjusting for two independent measures of illness severity (APACHE II and SOFA), arguing against simple severity confounding as the explanation. Because this pattern could only be observed by measuring the full axis simultaneously, it is a genuinely novel structural observation rather than an incremental replication of prior single-marker GDF-15 or IGFBP-2 studies.

One biologically plausible interpretation, among several, is that GDF-15 suppresses hepatic JAK2/STAT5 signaling downstream of the GH receptor [12,13], potentially altering IGF-1 bioavailability and binding proteins without necessarily affecting GHR expression. The clustering of GDF-15 with IGFBP-1, IGFBP-2, and GHBP, but not GHR, is compatible with—but does not establish—a model in which GDF-15 acts downstream of, or parallel to, receptor-level GH resistance, modulating IGF-1 bioavailability through binding-protein regulation rather than GHR expression. Importantly, hierarchical clustering identifies statistical co-variation, not a validated biological pathway; this mechanistic interpretation is based on the observed clustering and prior literature [12,13], not on the clustering itself. No functional or mechanistic experiments were performed, and the cross-sectional observational design cannot establish causality or temporal sequence. Direct mechanistic testing is therefore required.

A related question is whether this cluster reflects generic inflammatory burden rather than a specific GH–IGF-axis phenomenon. Sensitivity analyses in Section 3.7 provide a more precise answer: the GDF-15–IGFBP-1 and GDF-15–IGFBP-2 correlations remain essentially unchanged after adjustment for IL-6, arguing against a simple inflammatory-echo explanation, whereas the GDF-15–GHBP correlation attenuates substantially, suggesting that this association largely reflects shared IL-6-related inflammatory burden rather than a GDF-15-specific mechanism. Thus, the inhibitory-IGFBP component appears to be the more specific and robust feature of the cluster, with the GHBP association representing a weaker, more confounded satellite (Figure 6).

The retained correlation strength after adjustment for APACHE II and SOFA is, in our view, the most informative single result in this study. The two severity scores capture organ dysfunction (SOFA) and acute physiological derangement plus chronic health (APACHE II) through largely independent routes; that the GDF-15–IGFBP correlations were stable under both adjustments strengthens, though does not prove, the inference that a more specific shared regulatory pathway, rather than generic severity, underlies the association.

In secondary, explicitly exploratory analyses, GDF-15 also showed the strongest individual association with ICU mortality among the ten biomarkers (AUC 0.74), consistent with prior reports in heterogeneous ICU and sepsis populations [14,15]. Our GDF-15 AUC (0.74) sits within the range reported by larger ICU/sepsis cohorts examining GDF-15 or IGFBP-2 [14,15,19,20], and the trajectory-subtype pattern we observed is qualitatively consistent with multicenter longitudinal phenotyping work [16]; however, our point estimates carry wider uncertainty at n = 43 than those larger-cohort estimates, and direct numerical comparison should be made cautiously given differing case-mix, assay platforms, and endpoint definitions across studies. However, this discrimination was substantially weaker than that of APACHE II (0.90) or SOFA (0.81), and a combined GDF-15 + IGFBP-2 model did not improve on GDF-15 alone under leave-one-out cross-validation. The consistent finding that GDF-15 and IGFBP-2, individually or combined, do not approach the discrimination achieved by APACHE II or SOFA argues against any near-term role for these biomarkers as mortality-prediction tools in isolation; their potential value, if any, is more likely to lie in mechanistic stratification—for example, identifying patients with a specific GDF-15-driven inhibitory-IGFBP signature (Section 3.7, Figure 6)—than in outcome prediction, which is already well served by validated severity scores. We present these results only as context for the correlation findings, not as evidence of clinical utility: with 9 events, AUC estimates carry wide uncertainty, were not externally validated, and should not be used to inform clinical decisions.

The longitudinal analyses illustrate the limitations of this sample size. The exploratory GEE-approximation analysis (Supplementary Table S2, Supplementary Figure S1) suggested a persistently elevated GDF-15 trajectory in non-survivors, broadly consistent with trajectory-subtype findings in larger cohorts [16]. However, an assumption-light label-permutation test using pooled early-vs-late values found this direction was not stable (Section 3.6.2). We interpret this as direct empirical evidence that, at n = 9 non-survivors declining to n = 3 by the final timepoint, longitudinal claims from this dataset are unstable to reasonable analytic choices (but reported transparently).

A specific and legitimate concern is whether pooling brain-injury and non-brain-injury patients obscures diagnosis-specific biology, since acute brain injury elevates GDF-15 principally through a central catecholamine surge and direct tissue destruction, a mechanism distinct from the cytokine-driven route more typical of systemic sepsis. Our diagnosis-stratified sensitivity analysis (Section 3.7) shows that this concern is partly, but not uniformly, borne out: the GDF-15–IGFBP-1 relationship is consistent in both brain-injury and non-brain-injury strata, whereas GDF-15–IGFBP-2 and IGFBP-1–IGFBP-2 are essentially confined to the non-brain-injury stratum. Therefore, GDF-15–IGFBP-1 appear to be the more diagnosis-independent, and by extension the more generalizable, components. Consistent with this heterogeneity, when we examined the primary admission diagnoses of the 9 non-survivors as a proxy for cause of death (a formally adjudicated cause of death was not part of this study’s data collection), 5/9 had a primary neurological/traumatic diagnosis and 4/9 had a non-neurological, uniformly septic, presentation (Section 3.7)—two plausibly distinct trajectories to death that a pooled analysis cannot separate with only 9 events.

This study has several important limitations: (1) Sample size and event number: with 43 patients and 9 ICU deaths, every quantitative estimate in this study, including the principal correlation finding, is a pilot estimate with wide uncertainty; 45 pairwise correlations were tested, and although hierarchical clustering, partial-correlation adjustment, and Benjamini–Hochberg FDR correction (Section 3.7) mitigate but do not eliminate concern about multiple comparisons and false discovery. (2) Single-center design and external validation: this is a single-center cohort; clinical implementation of any findings presented in this manuscript should await independent replication in multicenter studies. (3) Diagnostic heterogeneity and selection: the study was done in an ICU that serves as a general ICU, 51% of the patients in the study’s cohort had brain injury, and our own diagnosis-stratified sensitivity analysis (Section 3.7) shows that the principal cluster was not uniformly present across brain-injury and non-brain-injury strata; in addition, our exclusion of ICU stays <3 days (needed to ensure ≥1 follow-up sample) and of patients started on corticosteroids before sampling could be completed may have under-represented the most rapidly fatal presentations. Thus, selection bias cannot be excluded. (4) Unmeasured clinical detail: several variables were not captured as structured fields in the original data-collection protocol and could not be adjusted for, including maximum vasopressor dose-intensity, mechanical-ventilation P/F ratio and PEEP, renal and hepatic function, and nutritional status; we adjusted for the clinical variables that were available (age, BMI, mechanical-ventilation duration, IL-6, APACHE II, SOFA; Section 3.7) but this adjustment set is not exhaustive. (5) Measurement platform and assay surrogate: single-center, assay-specific ELISA measurement limits external validity, and GHR expression was measured in peripheral blood mononuclear cells rather than hepatocytes, the primary site of GH-driven IGF-1 production in critical illness; PBMC GHR is a circulating-immune-cell surrogate that may not track hepatic receptor biology, which constrains interpretation of the classical GH-resistance markers specifically. (6) Outcome definition: ICU mortality was used as the primary endpoint (Section 2.1); a 28-day mortality sensitivity analysis (Section 3.7) was directionally consistent but weaker, and endpoint choice should be considered when interpreting the discrimination estimates. (7) Survivorship-driven attrition: declining sample size at later timepoints is a structural feature of ICU mortality studies rather than a correctable design flaw, but it limits longitudinal inference as discussed above. (8) Causal inference: as an observational study, no causal inference is possible regarding the relationship between GDF-15, IGFBPs, and outcome.

These limitations notwithstanding, the central observation—that GDF-15 occupies a distinct position within the GH–IGF axis, clustering with inhibitory IGFBPs rather than classical GH-resistance markers, independent of illness severity—is a structural finding that could not have been observed without simultaneous multi-marker measurement. It generates a specific, testable hypothesis for future, adequately powered, multicenter studies: that GDF-15 and inhibitory IGFBPs share upstream regulatory control distinct from canonical GHR-mediated resistance, and that this shared regulation, rather than either marker alone, may better characterize the metabolic stress response in critical illness.

Building on these findings, we see three concrete next steps. First, direct measurement of hepatic JAK2/STAT5 signaling activity relative to GDF-15 and IGFBP levels, in mechanistic or animal models, is needed to test the proposed downstream-of-GHR hypothesis directly rather than inferentially. Second, a multicenter, adequately powered replication cohort with pre-specified diagnosis-stratified analysis is needed, given the brain-injury/non-brain-injury heterogeneity demonstrated in Section 3.7. Third, IL-6 and procalcitonin should be included as co-measured covariates in any future GDF-15/IGFBP biomarker panel, given the partial IL-6-mediated confounding of the GDF-15–GHBP link reported here.

## 5. Conclusions

The findings reported here should not be translated into clinical practice without independent multicenter replication; the following summary should be read with that constraint in mind. In this prospective pilot study of 43 critically ill patients, simultaneous measurement of all major GH–IGF axis components and GDF-15 revealed that GDF-15 clusters with the inhibitory binding proteins IGFBP-1 and IGFBP-2, as well as GHBP, distinct from classical GH-resistance markers, and that this intercorrelation structure is essentially unchanged after adjusting for illness severity (APACHE II, SOFA). This structural pattern was made possible only by simultaneous multi-marker measurement, and it generates a specific hypothesis about shared upstream regulation of GDF-15 and inhibitory IGFBPs in critical illness that is independent of generic severity. Secondary, exploratory analyses found that GDF-15 alone had the best individual discrimination for ICU mortality among the ten biomarkers tested, but this was substantially weaker than established severity scores, and longitudinal trajectory findings were unstable under an alternative analytic approach. The findings of this study require prospective assessment in independent, adequately powered, multicenter cohorts before any clinical interpretation is warranted.

## Figures and Tables

**Table 1:** Baseline characteristics by ICU survival status.

Variable	All (n = 43)	Survivors (n = 34)	Non-Survivors (n = 9)	*p*-Value
***Continuous***				
Age (years)	58.0 (51.0–68.0)	55.5 (46.0–66.5)	64.0 (58.0–69.0)	0.151
BMI (kg/m^2^)	26.5 (25.2–28.8)	26.3 (24.9–28.9)	27.7 (26.1–28.4)	0.339
APACHE II	12.0 (8.0–17.5)	10.0 (7.0–15.5)	18.0 (17.0–25.0)	<0.001 *
SOFA at T01	7.0 (3.0–9.0)	6.0 (2.0–8.8)	9.0 (8.0–11.0)	0.005 *
Days on MV	10.0 (0.0–22.0)	6.0 (0.0–20.2)	19.0 (13.0–34.0)	0.007 *
***Categorical***				
Female sex	18 (42%)	16 (47%)	2 (22%)	0.263
Brain injury	22 (51%)	17 (50%)	5 (56%)	1.000
Sepsis	10 (23%)	6 (18%)	4 (44%)	0.177
MV at T01	30 (70%)	22 (65%)	8 (89%)	0.237
≥1 documented comorbidity	26 (60%)	18 (53%)	8 (89%)	0.065
Vasopressor at T01	29 (67%)	22 (65%)	7 (78%)	0.693
ICU death	9 (21%)	0 (0%)	9 (100%)	—
28-day death	5 (12%)	0 (0%)	5 (56%)	—

Values are reported as median (IQR) for continuous variables and n (%) for categorical variables. Mann–Whitney U test was used for continuous; Fisher’s exact for categorical. All *p*-values are exploratory and unadjusted for multiple comparisons. * Nominally significant (*p* < 0.05). MV = mechanical ventilation; APACHE II [22]; SOFA [23]. Comorbidity was assessed from the structured past-medical-history field recorded prospectively for each patient; a formal weighted comorbidity index was not constructed because the underlying entries were recorded as free-text rather than coded diagnoses (see Supplementary Table S3 for category breakdown). Vasopressor exposure (norepinephrine and/or vasopressin) was derived from the T01 ICU medication record; maximum dose-intensity was not captured as a structured field and is not reported.

**Table 2:** Admission (T01) biomarker concentrations by ICU survival status, with Hodges–Lehmann (H–L) shift estimates and dual significance testing.

Biomarker	Surv. Median (IQR)	n	Non-Surv. Median (IQR)	n	H–L Shift	95% CI (HL)	MWU *p*	Perm. *p*	AUC
***GH–IGF Axis***									
GH (µg/L)	1.0 (0.4–2.4)	34	0.8 (0.6–1.2)	9	−0.15	[−0.88, 0.44]	0.633	0.680	0.55
IGF-1 (µg/L)	85.0 (58.0–101.0)	33	72.0 (51.3–96.8)	8	−8.0	[−39.0, 30.0]	0.587	0.624	0.56
GHR vs. HC	1.7 (0.8–4.4)	32	2.1 (0.9–3.0)	9	−0.28	[−1.37, 1.42]	0.765	0.993	0.53
GHR vs. T01	1.1 (0.5–2.6)	31	1.2 (0.5–1.8)	9	−0.17	[−0.85, 0.84]	0.771	0.983	0.53
ALS (µg/mL)	3.84 (3.47–4.04)	33	3.72 (3.63–3.89)	9	−0.10	[−0.58, 0.25]	0.646	0.369	0.55
GHBP (pmol/L)	22.4 (18.0–30.2)	33	29.6 (18.6–34.5)	9	6.70	[−3.29, 51.03]	0.209	0.081	0.64
***IGFBPs and GDF-15***									
GDF-15 (ng/mL)	5.97 (2.14–11.78)	33	12.96 (5.34–31.16)	9	8.37	[0.63, 65.84]	0.030 *	0.027 *	0.74
IGFBP-1 (µg/L)	18.2 (3.0–33.2)	34	33.6 (6.2–36.2)	9	3.01	[−5.00, 19.31]	0.362	0.514	0.60
IGFBP-2 (µg/L)	1347 (965–1813)	34	2074 (1288–2640)	9	680.3	[−22.2, 1316.6]	0.054 ‡	0.059 ‡	0.71
IGFBP-3 (µg/L)	170.5 (126.6–207.7)	34	171.4 (133.4–221.3)	9	4.42	[−47.3, 52.0]	0.800	0.697	0.53

Values are median (IQR). H–L shift = Hodges–Lehmann estimator (median of all pairwise non-survivor − survivor differences), with 95% bootstrap percentile CI (3000 resamples); wide intervals reflect n = 9 non-survivors and should be read as a precision statement, not as evidence against an effect. MWU *p*: Mann–Whitney U. Perm. *p*: two-sided label-permutation test on log-transformed values (10,000 resamples), reported as a distribution-free cross-check of the MWU result. All *p*-values are unadjusted for multiple comparisons across 10 biomarkers; interpret as exploratory only. AUC: area under ROC curve for ICU mortality. * Nominally significant by both tests (unadjusted *p* < 0.05). ‡ Borderline trend by both tests. GHR vs. HC = GH receptor relative to healthy controls; GHR vs. T01 = relative to own admission value. ALS = acid-labile subunit; GDF-15 = growth differentiation factor 15; GHBP = GH-binding protein.

**Table 3:** Unadjusted and severity-adjusted (partial) Spearman correlations for key biomarker pairs identified by hierarchical clustering.

Biomarker Pair	ρ (Raw)	95% CI	ρ | APACHE	*p*	ρ | SOFA	*p*	n
GDF-15 vs. IGFBP-1	0.65	[0.44, 0.78]	0.67	<0.001	0.69	<0.001	42
GDF-15 vs. IGFBP-2	0.50	[0.19, 0.71]	0.50	<0.001	0.50	<0.001	42
GDF-15 vs. GHBP	0.47	[0.20, 0.68]	0.31	0.047	0.34	0.029	42
GDF-15 vs. IGF-1	−0.31	[−0.60, 0.04]	−0.34	0.034	−0.36	0.023	40
IGF-1 vs. IGFBP-2	−0.49	[−0.72, −0.18]	−0.49	0.001	−0.50	0.001	41
IGF-1 vs. IGFBP-1	−0.37	[−0.61, −0.08]	−0.37	0.018	−0.37	0.016	41
IGFBP-1 vs. IGFBP-2	0.45	[0.14, 0.69]	0.44	0.003	0.45	0.003	43

ρ (raw): unadjusted Spearman correlation; 95% CI: bootstrap percentile confidence interval (5000 resamples). ρ | APACHE, ρ | SOFA: partial Spearman correlation adjusting for APACHE II or SOFA respectively, computed via rank-residual regression. All *p*-values are exploratory and unadjusted for the 7 pairs shown. The consistently small change between raw and partial estimates (≤0.16 in all cases) indicates the structure is not primarily attributable to shared severity confounding.

**Table 4:** Spearman rank correlations between admission (T01) biomarkers and illness severity scores.

Biomarker	N	APACHE II ρ	APACHE II *p*	N	SOFA ρ	SOFA *p*	AUC
GH–IGF Axis							
GH	43	−0.128	0.412	43	−0.124	0.427	0.55
IGF-1	41	−0.044	0.786	41	0.008	0.958	0.56
GHR vs. HC	41	0.119	0.457	41	0.278	0.078	0.53
GHR vs. T01	40	0.124	0.447	40	0.275	0.086	0.53
ALS	42	−0.116	0.466	42	0.053	0.737	0.55
GHBP	42	0.476	0.001 *	42	0.452	0.003 *	0.64
IGFBPs and GDF-15							
GDF-15	42	0.501	0.001 *	42	0.455	0.002 *	0.74
IGFBP-1	43	0.142	0.365	43	0.096	0.540	0.60
IGFBP-2	43	0.102	0.514	43	0.086	0.585	0.71
IGFBP-3	43	−0.059	0.706	43	0.015	0.924	0.53

All *p*-values exploratory and unadjusted. * Nominally significant (*p* < 0.05). AUC: area under ROC curve for ICU mortality. Abbreviations as in Table 2.

**Table 5:** Sensitivity of the four key T01 biomarker correlations to extended confounder adjustment. Partial Spearman ρ shown for each adjustment set (rank-residual method, n = 40–43 per pair; see Supplementary Methods). * *p* < 0.05; ** *p* < 0.01; *** *p* < 0.001; ns = not significant (*p* ≥ 0.05). Diagnosis-stratified (brain-injury) sensitivity, Benjamini–Hochberg FDR q-values for all 45 pairwise T01 correlations, comorbidity and sepsis-source detail, and the non-survivor diagnosis-category breakdown are given in Supplementary Table S3.

Biomarker Pair	ρ (Raw)	ρ | +IL-6	ρ | +IL-6+APACHE	ρ | +Age+BMI	ρ | +MV days
GDF-15 vs. IGFBP-1	0.65 ***	0.53 ***	0.56 ***	0.70 ***	0.65 ***
GDF-15 vs. IGFBP-2	0.50 ***	0.56 ***	0.56 ***	0.44 **	0.45 **
GDF-15 vs. GHBP	0.47 **	0.24 (ns)	0.21 (ns)	0.51 ***	0.39 *
IGFBP-1 vs. IGFBP-2	0.45 **	0.46 **	0.47 **	0.49 ***	0.43 **

## Data Availability

Anonymized data used in this study are available at Zenodo: https://doi.org/10.5281/zenodo.20844938. During the preparation of this manuscript, the authors used Claude Sonnet 4.6 (Anthropic, https://claude.ai/, accessed on 1 May 2026) and Gemini 2.5 Flash (Google, https://gemini.google.com/, accessed on 1 May 2026) for statistical code assistance, artwork, and language/syntax corrections. The authors have reviewed and edited the output and take full responsibility for the content of this publication.

## Supplementary Materials

The following supporting information can be downloaded at: https://www.mdpi.com/article/10.3390/life16081245/s1, Supplementary Methods: full technical detail for statistical procedures summarized in Section 2.3 (resampling counts, rank-residual partial-correlation construction, GEE cluster-count rationale and variance-estimator specification, LOO-CV procedure); Supplementary Figure S1: Longitudinal trajectories of all ten biomarkers (T01–T04) with exploratory GEE-approximation p-values; Supplementary Table S1: Full descriptive statistics for all biomarkers at all timepoints; Supplementary Table S2: Full exploratory GEE-approximation results (group and group × time interaction terms) for all ten biomarkers, in-cluding a sensitivity analysis restricted to patients with ≥2 timepoints; Supplementary Table S3: Extended sensitivity analyses—Benjamini–Hochberg FDR q-values for all 45 pairwise T01 correla-tions, brain-injury-stratified sensitivity analysis, IL-6/procalcitonin/lactate correlations with cluster markers, comorbidity category summary, sepsis-source detail, non-survivor diagnosis-category breakdown, and 28-day mortality sensitivity analysis.

## Abbreviations

**Abbreviation**	**Full Term**
ALS	Acid-Labile Subunit
APACHE II	Acute Physiology and Chronic Health Evaluation II
AUC	Area Under the Curve
BMI	Body Mass Index
CI	Confidence Interval
CV	Coefficient of Variation
ELISA	Enzyme-Linked Immunosorbent Assay
FDR	False Discovery Rate
GDF-15	Growth Differentiation Factor 15
GEE	Generalized Estimating Equations
GH	Growth Hormone
GHBP	Growth Hormone-Binding Protein
GHR	Growth Hormone Receptor
GHR vs. HC	Growth Hormone Receptor Expression Relative to Healthy Controls
GHR vs. T01	Growth Hormone Receptor Expression Relative to Admission Value (T01)
GR	Glucocorticoid Receptor
ICU	Intensive Care Unit
IGF	Insulin-Like Growth Factor
IGF-1	Insulin-Like Growth Factor 1
IGFBP	Insulin-Like Growth Factor-Binding Protein
IGFBP-1	Insulin-Like Growth Factor-Binding Protein 1
IGFBP-2	Insulin-Like Growth Factor-Binding Protein 2
IGFBP-3	Insulin-Like Growth Factor-Binding Protein 3
IL	Interleukin
IL-1	Interleukin-1
IL-6	Interleukin-6
IQR	Interquartile Range
JAK2	Janus Kinase 2
LOO-CV	Leave-One-Out Cross-Validation
MV	Mechanical Ventilation
MWU	Mann–Whitney U (test)
PBMC	Peripheral Blood Mononuclear Cell
PCT	Procalcitonin
ROC	Receiver Operating Characteristic
RT-PCR	Reverse Transcription Polymerase Chain Reaction
SOCS3	Suppressor of Cytokine Signaling 3
SOFA	Sequential Organ Failure Assessment
STAT5	Signal Transducer and Activator of Transcription 5
STROBE	Strengthening the Reporting of Observational Studies in Epidemiology
T01	Admission Timepoint (within 48 h of ICU admission)
T02	Day 5 ± 1 Timepoint
T03	Day 10 ± 1 Timepoint
T04	Day 15 ± 1 Timepoint
TGF-β	Transforming Growth Factor Beta
TNF	Tumor Necrosis Factor
TNF-α	Tumor Necrosis Factor Alpha
