# Supplementary material for: Growth Hormone–Insulin-like Growth Factor Axis and GDF-15 in Critical Illness: Implications for Survival Stratification
Source: Life (Basel). 2026 Jul 27;16(8):1245. doi: 10.3390/life16081245 (PMC13514936; doi:10.3390/life16081245)
Supplement: Supplementary file 1 [file life-16-01245-s001.zip › life-4426314-supplementary.pdf]

# Methods

*This section provides full technical detail for the statistical procedures summarized in Section 2.3 of the main text, added in response to reviewer requests to shorten the main-text Methods.*

**Partial correlation construction (rank-residual method).**

For each pair of biomarkers (X, Y) and covariate set Z, all variables were rank-transformed (Spearman ranks). Ranks of X and of Y were each regressed on the ranks of the covariates in Z (ordinary least squares, with an intercept); the Pearson correlation of the two sets of residuals gives the partial Spearman correlation of X and Y adjusting for Z. This rank-residual approach avoids distributional assumptions beyond monotonicity and is robust to the small, non-normally-distributed biomarker values in this cohort.

**Bootstrap confidence intervals.**

95% confidence intervals for key Spearman correlation estimates were constructed using the percentile bootstrap with 5000 resamples: patients were resampled with replacement, the correlation recomputed in each resample, and the 2.5th and 97.5th percentiles of the resulting distribution taken as the interval bounds.

**Benjamini–Hochberg false discovery rate correction.**

Across the 45 pairwise Spearman correlations computed among the ten T01 biomarkers, raw p-values were ranked from smallest to largest (rank $i = 1...45$). The Benjamini–Hochberg critical value for each rank was $p(i) \times 45 / i$; q-values were computed as the minimum of this quantity over all ranks $\geq i$ (ensuring monotonicity), capped at 1. Pairs with $q < 0.05$ were considered FDR-significant. Full q-values for all 45 pairs are given in Supplementary Table S3.

**GEE-approximation cluster-count rationale and variance-estimator specification.**

Longitudinal biomarker trajectories (T01–T04) were modelled using a generalized-estimating-equations approximation with an independence working correlation structure and a cluster-robust (sandwich) variance estimator, clustered by patient, applied to log-transformed biomarker values with fixed effects for survival group, time, and their interaction. Reliable cluster-robust sandwich variance estimation generally requires approximately ≥30–40 independent clusters for the sandwich estimator's asymptotic properties to hold; the non-survivor group in this cohort contributes only 9 clusters at T01, declining to 3 clusters by T04, far below this threshold. Consequently, GEE p-values for the non-survivor group, and any group×time interaction terms involving it, are reported in Supplementary Table S2 for transparency but should not be interpreted as reliable inferential statistics; they are exploratory descriptive signals only. A sensitivity analysis restricting the primary model to patients contributing ≥2 timepoints (reducing informative-missingness bias from single-timepoint patients) is reported alongside the primary model in Supplementary Table S2.

**Leave-one-out cross-validation (LOO-CV) procedure.**

For each of the $n = 43$ patients in turn, a logistic regression model (for single biomarkers: GDF-15 or IGFBP-2, log-transformed and standardized; for the combined model: both markers jointly) was fit to the remaining $n-1$ patients and used to predict the held-out patient's probability of ICU death. The area under the ROC curve (AUC) was then computed from the $n$ out-of-sample predicted probabilities and the observed outcomes. This procedure avoids the optimistic bias of in-sample AUC estimation but, at $n = 43$ with 9 events, still carries substantial sampling variability not captured by the point estimate alone.

**Brain-injury-stratified sensitivity analysis and comorbidity/sepsis-source ascertainment.**
The diagnosis-stratified sensitivity analysis (Section 3.7) repeated the four key T01 Spearman correlations separately within the brain-injury (n = 22) and non-brain-injury (n = 21) subgroups defined by primary admission diagnosis. Comorbidity, vasopressor exposure, and sepsis-source information (Supplementary Table S3) were derived from structured review of the prospectively recorded clinical fields (past-medical-history free text and ICU medication record); no new clinical data were collected retrospectively beyond what was already part of the original study record.

## Supplementary Figure S1

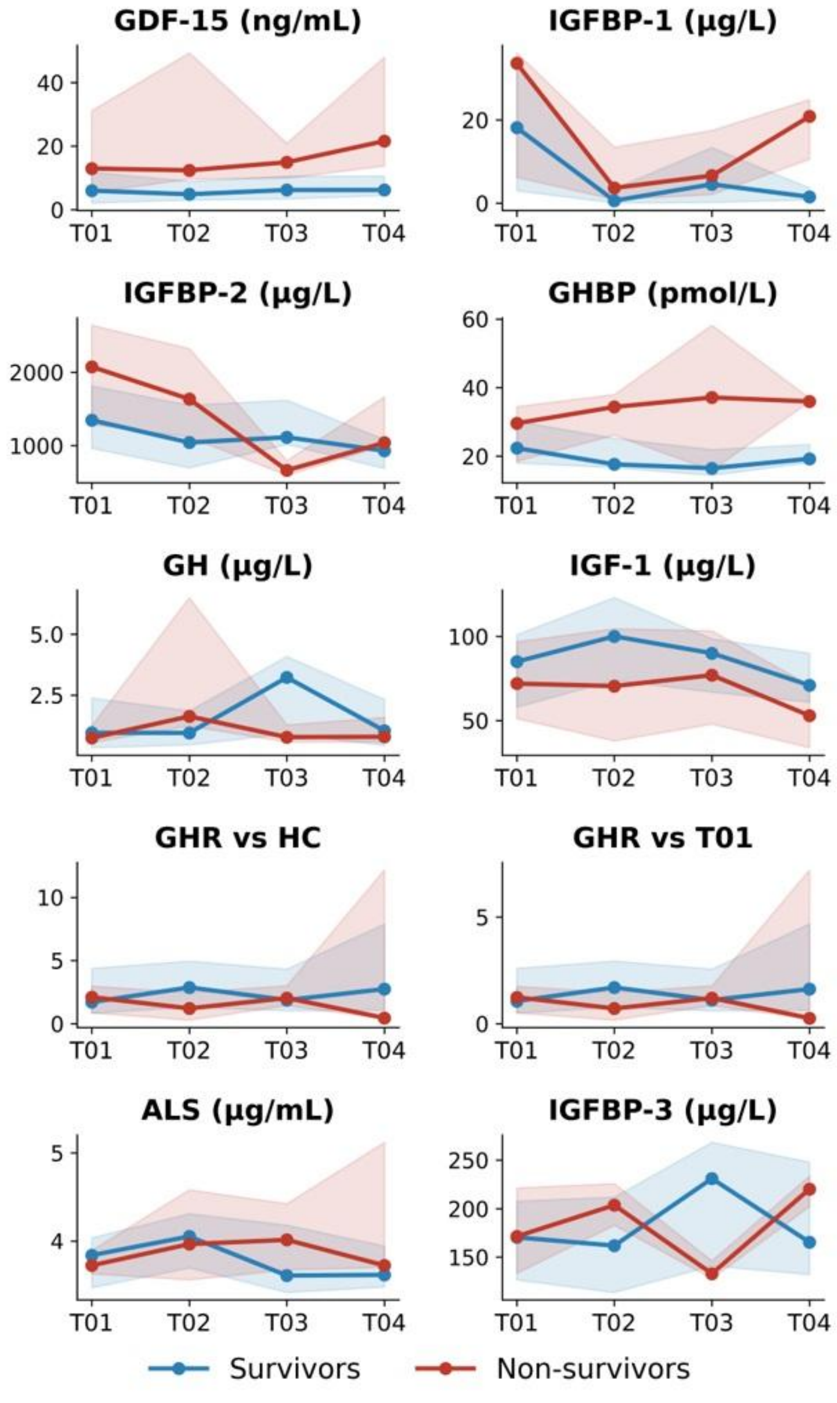


*Longitudinal biomarker trajectories (T01–T04) by ICU survival status, for all ten GH–IGF axis / GDF-15 biomarkers. Median (line) and IQR (shaded band) at each timepoint; underlying numeric values are given in Supplementary Table S1. Sample sizes decline across timepoints owing to ICU discharge (survivors) and death (non-survivors): T01 n=43, T02 n=28, T03 n=19, T04 n=11 (see Figure 1). Group and group×time interaction p-values from the exploratory GEE-approximation model are reported in Supplementary Table S2. This figure was previously described in the main text but not rendered; it is renumbered from Supplementary Figure S2 to S1 following promotion of the STROBE flow diagram to main-text Figure 1.*

## Supplementary Table S1

Descriptive statistics for all biomarkers at each timepoint, by ICU survival status.

**T01 (Day ~1)**

| | Survivors (n = 34) | | | | Non-survivors (n = 9) | | | | |
|---|---|---|---|---|---|---|---|---|---|
| **Biomarker** | **n** | **Q1** | **Median** | **Q3** | **n** | **Q1** | **Median** | **Q3** | **MWU p** |
| GH (μg/L) | 34 | 0.35 | **0.97** | 2.39 | 9 | 0.57 | **0.75** | 1.25 | 0.633 |
| IGF-1 (μg/L) | 33 | 58.0 | **85.0** | 101.0 | 8 | 51.3 | **72.0** | 96.8 | 0.587 |
| GHR vs HC | 32 | 0.83 | **1.71** | 4.37 | 9 | 0.86 | **2.10** | 2.96 | 0.765 |
| GHR vs T0 | 31 | 0.50 | **1.06** | 2.59 | 9 | 0.51 | **1.24** | 1.75 | 0.771 |
| ALS (μg/mL) | 33 | 3.47 | **3.84** | 4.04 | 9 | 3.63 | **3.72** | 3.89 | 0.646 |
| GHBP (pmol/L) | 33 | 18.0 | **22.4** | 30.2 | 9 | 18.6 | **29.6** | 34.5 | 0.209 |
| GDF-15 (ng/mL) | 33 | 2.14 | **5.97** | 11.78 | 9 | 5.34 | **12.96** | 31.16 | **0.030** |
| IGFBP-1 (μg/L) | 34 | 3.0 | **18.2** | 33.2 | 9 | 6.2 | **33.6** | 36.2 | 0.362 |
| IGFBP-2 (μg/L) | 34 | 965 | **1347** | 1813 | 9 | 1288 | **2074** | 2640 | 0.054 |
| IGFBP-3 (μg/L) | 34 | 126.6 | **170.5** | 207.7 | 9 | 133.4 | **171.4** | 221.3 | 0.800 |

**T02 (Day ~5)**

| | Survivors (n = 34) | | | | Non-survivors (n = 9) | | | | |
|---|---|---|---|---|---|---|---|---|---|
| **Biomarker** | **n** | **Q1** | **Median** | **Q3** | **n** | **Q1** | **Median** | **Q3** | **MWU p** |
| GH (μg/L) | 22 | 0.47 | **0.95** | 1.86 | 6 | 1.24 | **1.64** | 6.50 | 0.300 |
| IGF-1 (μg/L) | 21 | 74.0 | **100.0** | 123.0 | 6 | 38.0 | **70.5** | 104.5 | 0.145 |
| GHR vs HC | 22 | 1.31 | **2.87** | 4.95 | 6 | 0.29 | **1.21** | 2.48 | 0.214 |
| GHR vs T0 | 22 | 0.77 | **1.70** | 2.94 | 6 | 0.17 | **0.71** | 1.46 | 0.214 |
| ALS (μg/mL) | 21 | 3.70 | **4.05** | 4.31 | 6 | 3.56 | **3.96** | 4.58 | 0.953 |
| GHBP (pmol/L) | 21 | 16.7 | **17.6** | 25.2 | 6 | 26.1 | **34.4** | 38.0 | **0.033** |
| GDF-15 (ng/mL) | 21 | 3.06 | **4.85** | 8.92 | 6 | 9.68 | **12.39** | 49.38 | **0.011** |
| IGFBP-1 (μg/L) | 22 | 0.1 | **0.6** | 3.5 | 6 | 0.9 | **3.7** | 13.5 | 0.133 |
| IGFBP-2 (μg/L) | 22 | 698 | **1043** | 1551 | 6 | 1118 | **1635** | 2321 | 0.078 |
| IGFBP-3 (μg/L) | 22 | 113.7 | **161.9** | 211.9 | 6 | 182.9 | **203.6** | 225.8 | 0.175 |

**T03 (Day ~10)**

| | Survivors (n = 34) | | | | Non-survivors (n = 9) | | | | |
|---|---|---|---|---|---|---|---|---|---|
| **Biomarker** | **n** | **Q1** | **Median** | **Q3** | **n** | **Q1** | **Median** | **Q3** | **MWU p** |
| GH (μg/L) | 15 | 0.93 | **3.23** | 4.08 | 4 | 0.59 | **0.79** | 1.28 | 0.124 |
| IGF-1 (μg/L) | 15 | 67.0 | **90.0** | 98.5 | 4 | 48.0 | **77.0** | 103.3 | 0.548 |
| GHR vs HC | 15 | 1.02 | **1.87** | 4.32 | 4 | 1.44 | **2.03** | 3.00 | 0.960 |
| GHR vs T0 | 15 | 0.61 | **1.11** | 2.56 | 4 | 0.85 | **1.21** | 1.78 | 0.960 |

| | | | | | | | | | |
|---|---|---|---|---|---|---|---|---|---|
| ALS (μg/mL) | 14 | 3.42 | **3.61** | 4.18 | 4 | 3.67 | **4.01** | 4.42 | 0.505 |
| GHBP (pmol/L) | 14 | 14.4 | **16.5** | 21.9 | 4 | 15.8 | **37.1** | 58.2 | 0.233 |
| GDF-15 (ng/mL) | 14 | 3.40 | **6.21** | 10.54 | 4 | 9.96 | **14.92** | 20.90 | 0.277 |
| IGFBP-1 (μg/L) | 15 | 0.2 | **4.6** | 13.4 | 4 | 2.1 | **6.7** | 17.5 | 0.801 |
| IGFBP-2 (μg/L) | 15 | 1018 | **1114** | 1618 | 4 | 597 | **665** | 796 | **0.037** |
| IGFBP-3 (μg/L) | 15 | 141.1 | **231.1** | 268.4 | 4 | 128.0 | **133.0** | 146.2 | 0.100 |

**T04 (Day ~15)**

| | Survivors (n = 34) | | | | Non-survivors (n = 9) | | | | |
|---|---|---|---|---|---|---|---|---|---|
| **Biomarker** | **n** | **Q1** | **Median** | **Q3** | **n** | **Q1** | **Median** | **Q3** | **MWU p** |
| GH (μg/L) | 8 | 0.45 | **1.04** | 2.32 | 3 | 0.59 | **0.80** | 1.59 | 1.000 |
| IGF-1 (μg/L) | 8 | 61.0 | **71.0** | 90.0 | 3 | 34.0 | **53.0** | 71.0 | 0.306 |
| GHR vs HC | 8 | 1.08 | **2.74** | 7.89 | 3 | 0.40 | **0.45** | 12.17 | 0.474 |
| GHR vs T0 | 8 | 0.64 | **1.62** | 4.68 | 3 | 0.23 | **0.26** | 7.21 | 0.474 |
| ALS (μg/mL) | 7 | 3.48 | **3.62** | 3.94 | 3 | 3.70 | **3.72** | 5.12 | 0.383 |
| GHBP (pmol/L) | 7 | 18.1 | **19.2** | 23.4 | 3 | 35.9 | **36.0** | 36.9 | **0.017** |
| GDF-15 (ng/mL) | 7 | 4.47 | **6.20** | 10.61 | 3 | 13.75 | **21.59** | 48.05 | 0.183 |
| IGFBP-1 (μg/L) | 8 | 0.8 | **1.5** | 3.7 | 3 | 10.6 | **20.9** | 24.9 | 0.279 |
| IGFBP-2 (μg/L) | 8 | 690 | **930** | 1083 | 3 | 1002 | **1040** | 1665 | 0.376 |
| IGFBP-3 (μg/L) | 8 | 132.1 | **165.4** | 248.0 | 3 | 202.2 | **220.2** | 233.7 | 0.630 |

*Values are n (available), Q1 (25th percentile), median (50th percentile, bold), and Q3 (75th percentile). MWU p: two-sided Mann–Whitney U test at each timepoint, unadjusted for multiple comparisons; all p-values exploratory. Patient counts at T02–T04 reflect those still in ICU with available samples; attrition is primarily due to ICU discharge (survivors) and death (non-survivors), introducing informative missingness at later timepoints. GHR vs HC = GH receptor relative to healthy controls; GHR vs T0 = relative to admission. ALS = acid-labile subunit; GDF-15 = growth differentiation factor 15; GHBP = GH-binding protein.*

## Supplementary Table S2

Full exploratory GEE-approximation results (group main effect and group×time interaction) for all ten biomarkers, including a sensitivity analysis restricted to patients contributing data at ≥2 timepoints. All p-values in this table are exploratory and hypothesis-generating only; they should not be interpreted as confirmatory given the very small and declining non-survivor cluster count described in the main text (Section 2.3).

| | **Primary (all available data)** | | | | **Sensitivity (≥2 timepoints)** | | | |
|---|---|---|---|---|---|---|---|---|
| **Biomarker** | **p (group)** | **p (time×group)** | **Clusters** | **NS clusters** | **p (group)** | **p (time×group)** | **Clusters** | **NS clusters** |
| ***GH–IGF Axis*** | | | | | | | | |
| GH | 0.962 | 0.658 | 43 | 9 | 0.264 | 0.232 | 28 | 6 |
| IGF-1 | 0.572 | 0.242 | 41 | 8 | 0.352 | 0.509 | 27 | 6 |
| GHR vs HC | 0.657 | 0.583 | 41 | 9 | 0.864 | 0.626 | 27 | 6 |
| GHR vs T01 | 0.674 | 0.564 | 40 | 9 | 0.828 | 0.647 | 27 | 6 |
| ALS | 0.367 | 0.123 | 42 | 9 | 0.169 | 0.103 | 27 | 6 |
| GHBP | 0.111 | 0.338 | 42 | 9 | 0.525 | 0.255 | 27 | 6 |
| ***IGFBPs and GDF-15*** | | | | | | | | |
| GDF-15 | 0.007 | 0.447 | 42 | 9 | 0.006 | 0.170 | 27 | 6 |
| IGFBP-1 | 0.342 | 0.577 | 43 | 9 | 0.474 | 0.681 | 28 | 6 |
| IGFBP-2 | 0.037 | 0.137 | 43 | 9 | 0.007 | 0.020 | 28 | 6 |
| IGFBP-3 | 0.920 | 0.965 | 43 | 9 | 0.859 | 0.930 | 28 | 6 |

*Primary model: exploratory GEE-approximation with cluster-robust sandwich variance (clustered by patient), fit to log-transformed biomarker values across all available T01–T04 observations, using all enrolled patients. Sensitivity model: identical specification restricted to patients contributing data at ≥2 of the four timepoints, reducing total clusters but partially addressing single-timepoint instability. NS clusters: number of non-survivor patients contributing to the model (clusters), which directly determines the reliability of cluster-robust variance estimation; reliable estimation generally requires 30–40+ clusters per group, far exceeding what either model achieves for the non-survivor group. All p-values exploratory, unadjusted for 10 simultaneous biomarker models, and not intended for confirmatory inference. Abbreviations as in main text Table 2.*

# Supplementary Table S3

*Extended sensitivity analyses: Benjamini–Hochberg FDR q-values for all 45 pairwise T01 correlations (S3a); brain-injury-stratified sensitivity analysis for the four key biomarker pairs (S3b); correlations of cluster markers with IL-6, procalcitonin, and lactate at T01 (S3c); comorbidity category summary (S3d); sepsis source detail (S3e); non-survivor primary-diagnosis-category breakdown, a proxy for cause of death since formal adjudication was not part of this study's data collection (S3f); and 28-day mortality sensitivity analysis for GDF-15 (S3g).*

## S3a. Benjamini–Hochberg FDR-corrected q-values, all 45 pairwise T01 correlations

| Biomarker pair | Spearman ρ | p (raw) | q (BH-FDR) | n |
|---|---|---|---|---|
| GHR vs HC vs. GHR vs T01 | 0.99 | 0.0000 | 0.0000* | 40 |
| GDF-15 vs. IGFBP-1 | 0.65 | 0.0000 | 0.0001* | 42 |
| IGF-1 vs. IGFBP-3 | 0.55 | 0.0002 | 0.0026* | 41 |
| GDF-15 vs. IGFBP-2 | 0.49 | 0.0009 | 0.0094* | 42 |
| IGF-1 vs. IGFBP-2 | -0.49 | 0.0010 | 0.0094* | 41 |
| GHBP vs. GDF-15 | 0.47 | 0.0016 | 0.0116* | 42 |
| IGFBP-1 vs. IGFBP-2 | 0.45 | 0.0024 | 0.0151* | 43 |
| ALS vs. IGFBP-3 | 0.45 | 0.0027 | 0.0154* | 42 |
| GH vs. IGFBP-1 | 0.40 | 0.0078 | 0.0392* | 43 |
| IGF-1 vs. IGFBP-1 | -0.37 | 0.0175 | 0.0787 | 41 |
| GHBP vs. IGFBP-1 | 0.34 | 0.0277 | 0.1134 | 42 |
| IGF-1 vs. GDF-15 | -0.31 | 0.0527 | 0.1976 | 40 |
| GH vs. ALS | 0.26 | 0.0990 | 0.3426 | 42 |
| GHR vs HC vs. IGFBP-2 | -0.25 | 0.1116 | 0.3589 | 41 |
| GH vs. GDF-15 | 0.23 | 0.1359 | 0.4077 | 42 |
| GHR vs T01 vs. IGFBP-2 | -0.22 | 0.1729 | 0.4863 | 40 |
| GHR vs T01 vs. ALS | 0.21 | 0.2072 | 0.5485 | 39 |
| GH vs. IGFBP-2 | 0.18 | 0.2583 | 0.6059 | 43 |
| IGFBP-2 vs. IGFBP-3 | -0.18 | 0.2583 | 0.6059 | 43 |
| IGF-1 vs. GHR vs HC | 0.18 | 0.2693 | 0.6059 | 39 |
| IGFBP-1 vs. IGFBP-3 | -0.16 | 0.2970 | 0.6232 | 43 |
| GHR vs HC vs. IGFBP-3 | 0.16 | 0.3075 | 0.6232 | 41 |
| GHR vs HC vs. ALS | 0.16 | 0.3224 | 0.6232 | 40 |
| GDF-15 vs. IGFBP-3 | -0.15 | 0.3324 | 0.6232 | 42 |
| IGF-1 vs. GHR vs T01 | 0.15 | 0.3650 | 0.6570 | 38 |
| GH vs. IGFBP-3 | 0.13 | 0.3896 | 0.6743 | 43 |
| IGF-1 vs. GHBP | 0.13 | 0.4131 | 0.6885 | 40 |
| ALS vs. GHBP | -0.11 | 0.4986 | 0.7920 | 42 |
| GHR vs T01 vs. IGFBP-3 | 0.10 | 0.5195 | 0.7920 | 40 |
| GHR vs HC vs. IGFBP-1 | -0.10 | 0.5331 | 0.7920 | 41 |
| GHBP vs. IGFBP-2 | 0.09 | 0.5500 | 0.7920 | 42 |
| GH vs. GHR vs HC | -0.09 | 0.5632 | 0.7920 | 41 |
| GH vs. GHR vs T01 | -0.07 | 0.6457 | 0.8788 | 40 |
| GH vs. GHBP | -0.07 | 0.6639 | 0.8788 | 42 |
| GHBP vs. IGFBP-3 | 0.06 | 0.7087 | 0.8984 | 42 |
| GHR vs T01 vs. GDF-15 | 0.06 | 0.7364 | 0.8984 | 39 |
| ALS vs. IGFBP-1 | -0.05 | 0.7460 | 0.8984 | 42 |
| GHR vs T01 vs. IGFBP-1 | -0.05 | 0.7586 | 0.8984 | 40 |
| IGF-1 vs. ALS | 0.02 | 0.8808 | 0.9754 | 40 |

| | | | | |
|---|---|---|---|---|
| GHR vs HC vs. GDF-15 | 0.02 | 0.8837 | 0.9754 | 40 |
| GH vs. IGF-1 | 0.02 | 0.9100 | 0.9754 | 41 |
| ALS vs. IGFBP-2 | -0.02 | 0.9104 | 0.9754 | 42 |
| GHR vs T01 vs. GHBP | -0.00 | 0.9893 | 0.9968 | 39 |
| ALS vs. GDF-15 | 0.00 | 0.9923 | 0.9968 | 42 |
| GHR vs HC vs. GHBP | 0.00 | 0.9968 | 0.9968 | 40 |

** q < 0.05 (FDR-significant). Pairs sorted by ascending raw p-value.*

## S3b. Diagnosis-stratified (brain-injury vs. non-brain-injury) sensitivity analysis

| Biomarker pair | ρ (brain injury) | p (brain injury) | n | ρ (non-brain injury) | p (non-brain injury) | n |
|---|---|---|---|---|---|---|
| GDF-15 vs. IGFBP-1 | 0.75 | 0.0001 | 21 | 0.63 | 0.0024 | 21 |
| GDF-15 vs. IGFBP-2 | 0.29 | 0.1966 | 21 | 0.63 | 0.0024 | 21 |
| GDF-15 vs. GHBP | 0.53 | 0.0132 | 21 | 0.39 | 0.0818 | 21 |
| IGFBP-1 vs. IGFBP-2 | 0.14 | 0.5354 | 22 | 0.48 | 0.0274 | 21 |

*Brain injury n = 22 (21 with complete pairwise data for most markers); non-brain-injury n = 21. The GDF-15–IGFBP-1 relationship is consistent across strata; GDF-15–IGFBP-2 and IGFBP-1–IGFBP-2 are essentially confined to the non-brain-injury stratum. See Section 3.7 and Discussion.*

## S3c. Correlations of cluster markers with IL-6, procalcitonin, and lactate (T01)

| Marker | ρ IL-6 | p | n | ρ PCT | p | n | ρ Lactate | p | n |
|---|---|---|---|---|---|---|---|---|---|
| GDF-15 | 0.76 | <0.0001 | 41 | 0.45 | 0.0103 | 32 | 0.24 | 0.1312 | 42 |
| IGFBP-1 | 0.44 | 0.0039 | 42 | 0.48 | 0.0059 | 32 | 0.34 | 0.0271 | 43 |
| IGFBP-2 | 0.15 | 0.3470 | 42 | 0.50 | 0.0037 | 32 | -0.01 | 0.9266 | 43 |
| GHBP | 0.49 | 0.0012 | 41 | 0.35 | 0.0525 | 32 | 0.22 | 0.1592 | 42 |

*PCT = procalcitonin (available for a subset, n = 32, owing to selective ordering in clinical practice); Lactate = maximum T01 lactate. GDF-15 correlates strongly with IL-6 and moderately with PCT but not with lactate. See Section 3.7 and Discussion ("echo" question).*

## S3d. Comorbidity category summary (from structured past-medical-history review)

| Category | n | % of cohort (n=43) |
|---|---|---|
| Alcohol/substance use disorder | 5 | 12% |
| Hypothyroidism / autoimmune thyroid disease | 5 | 12% |
| Psychiatric illness | 4 | 9% |
| Prior cerebrovascular disease | 3 | 7% |
| Cardiac disease (atrial fibrillation, mechanical valve) | 2 | 5% |
| Malignancy (prior) | 2 | 5% |
| Other (single instances: polycystic kidney disease, immune thrombocytopenic purpura, prior bariatric surgery, nephrolithiasis, osteoporosis, anemia) | 6 | 14% |
| No documented comorbidity | 17 | 40% |

*Categories are not mutually exclusive (patients may have ≥1 comorbidity); 26/43 (60%) had ≥1 documented comorbidity (Table 1). No formal weighted comorbidity index (e.g., Charlson) was constructed, as underlying entries are free-text rather than coded diagnoses.*

## S3e. Sepsis source detail (of 10 patients with sepsis)

| Source | n |
|---|---|
| Respiratory | 1 |
| Intra-abdominal | 1 |

| | |
|---|---|
| Cholecystitis | 1 |
| Bacteremia (Staphylococcus aureus, with respiratory co-source) | 1 |
| Source not systematically documented | 6 |

*A documented infection source was available for 4/10 sepsis patients. Systematic screening for infective endocarditis was not part of this study's protocol; a cohort-wide rate cannot be reported.*

## S3f. Non-survivor primary admission diagnosis category (proxy for cause of death, n = 9)

| Category | n | % of non-survivors |
|---|---|---|
| Neurological / traumatic (subarachnoid or intraventricular hemorrhage, or polytrauma with head injury) | 5 | 56% |
| Non-neurological, with documented sepsis during ICU stay (heat stroke with cardiac arrest, fever of unknown origin evolving to sepsis, and two further febrile/septic presentations) | 4 | 44% |

*A formally adjudicated proximate cause of death (e.g., by mortality review committee) was not part of this study's data collection and was not reconstructed retrospectively; this table reports primary admission diagnosis category as a data-supported proxy only. See Section 3.7 and Discussion.*

## S3g. 28-day mortality sensitivity analysis for GDF-15

| Endpoint | Events | GDF-15 AUC | Mann–Whitney p |
|---|---|---|---|
| ICU mortality (primary endpoint) | 9 | 0.74 | 0.030 |
| 28-day mortality (sensitivity endpoint) | 5 | 0.65 | 0.277 |

*28-day mortality analysis is directionally consistent with, but weaker than, the primary ICU-mortality analysis, as expected with fewer and more temporally distant events. See Section 2.1 (endpoint justification) and Section 3.7.*
